# Time‑resolved multi-omic analysis of paclitaxel exposure in human iPSC‑derived sensory neurons unveils mechanisms of chemotherapy‑induced peripheral neuropathy

**DOI:** 10.1038/s41419-026-08445-2

**Published:** 2026-02-10

**Authors:** Christian Schinke, Smilla K. Maierhof, Lois Hew, Valeria Fernandez Vallone, Silke Frahm, Narasimha Swamy Telugu, Sebastian Diecke, Andranik Ivanov, Richard Kovács, Dieter Beule, Marieluise Kirchner, Philipp Mertins, Ulrike Brüning, Jennifer A. Kirwan, Harald Stachelscheid, Matthias Endres, Petra Huehnchen, Wolfgang Boehmerle

**Affiliations:** 1 Charité—Universitätsmedizin Berlin, corporate member of Freie Universität Berlin and Humboldt-Universität zu Berlin, Klinik und Hochschulambulanz für Neurologie, Berlin, Germany; 2 Berlin Institute of Health at Charité, Universitätsmedizin Berlin, Berlin, Germany; 3 Charité—Universitätsmedizin Berlin, corporate member of Freie Universität Berlin, Humboldt-Universität zu Berlin, and Berlin Institute of Health, Einstein Center for Neurosciences Berlin, Berlin, Germany; 4 Berlin Institute of Health at Charité—Universitätsmedizin Berlin, Core Unit Pluripotent Stem Cells and Organoids (CUSCO), Berlin, Germany; 5 Max Delbrück Center for Molecular Medicine in the Helmholtz Association (MDC), Berlin, Germany; 6 Berlin Institute of Health at Charité—Universitätsmedizin Berlin, Core Unit Bioinformatics, Berlin, Germany; 7 Charité—Universitätsmedizin Berlin, corporate member of Freie Universität Berlin, Humboldt-Universität zu Berlin, Institut für Neurophysiologie, Berlin, Germany; 8 Berlin Institute of Health at Charité, Universitätsmedizin Berlin, Max Delbrück Center for Molecular Medicine (MDC), Berlin, Germany; 9 Metabolomics, Berlin Institute of Health at Charité-Universitätsmedizin Berlin, Berlin, Germany; 10 University of Veterinary Medicine, Vienna, Wien, Austria; 11 Charité—Universitätsmedizin Berlin, corporate member of Freie Universität Berlin, Humboldt-Universität zu Berlin, Center for Stroke Research Berlin, Berlin, Germany; 12 German Center for Neurodegenerative Diseases (DZNE), Berlin, Germany; 13 German Center for Cardiovascular Research (DZHK), Berlin, Germany; 14 German Center for Mental Health (DZPG), Berlin, Germany; 15 Charité—Universitätsmedizin Berlin, corporate member of Freie Universität Berlin, Humboldt-Universität zu Berlin, NeuroCure Cluster of Excellence, Berlin, Germany

**Keywords:** Experimental models of disease, Diseases of the nervous system, Mechanisms of disease

## Abstract

The microtubule-stabilizing drug paclitaxel remains the standard of care for various solid malignancies but frequently leads to chemotherapy-induced peripheral neuropathy (CIPN). CIPN is a leading cause for premature treatment termination and a significantly reduced quality of life in long-term cancer survivors. The molecular mechanisms of neuro-axonal degeneration, neuroinflammation, and pain in patients treated with paclitaxel remain incompletely understood, and there are currently no predictive biomarkers or preventive treatments. We used human iPSC-derived sensory neurons exposed to paclitaxel to comprehensively model the pathophysiology of CIPN. Neurotoxicity was assessed over time using viability assays and sequential RNA sequencing, as well as deep proteome and lipidomic analyses. We observed a time and dose-dependent decline of cell viability at clinically relevant paclitaxel doses. Sequential RNA sequencing defined *JUN* as an early immediate gene, followed by the overexpression of genes of the neuronal stress response (e.g., *ARID5A, WEE1*, *DUSP16, GADD45A*), neuronal injury and apoptotic pathways (e.g., *ATF3*, *HRK, BBC3 [PUMA]*, *BCL2L11 [BIM]*, *CASP3*), neuroinflammation and nociception (*CALCB*, *MMP10*, *IL31RA*, *CYSLTR2*, *C3AR1, TNFRSF12A*) and neuronal transduction (e.g., *CAMK2A*, *STOML3, PIRT*), while key enzymes of lipid biosynthesis were markedly downregulated (e.g., *LSS, HMGCS1*, *HMGCR*, *DHCR24*). Deep proteome analyses following 48 h of exposure to 100 nM paclitaxel revealed a strong correlation of differentially expressed RNA with proteins, and a marked degradation of essential axonal transport proteins such as kinesins, stathmins, and scaffold proteins. Consistent with the downregulation of rate-limiting enzymes of lipid biosynthesis, lipidome analysis confirmed deregulation of neuronal lipid homeostasis. In summary, paclitaxel induces transcriptomic and proteomic signatures of the neuronal stress response, neuroinflammation, nociception, and disturbed metabolism. These may explain, in part, the clinical phenotype of sensory loss, hypersensitivity, and neuropathic pain frequently observed in patients suffering from CIPN, but constitute pharmacologically addressable targets.

## Introduction

Chemotherapy-induced polyneuropathy (CIPN) affects about two-thirds of patients treated with neurotoxic antineoplastic therapy [1], frequently leading to dose reduction, early treatment termination, and potentially irreversible neurologic sequelae with significantly reduced quality of life in a growing number of long-term survivors [2–4]. Despite recent advances in cancer immune therapy [5], paclitaxel remains pivotal in the standard of care of various solid malignancies such as breast, lung, gastrointestinal, head and neck, or ovarian cancer, with millions of treated patients annually [6]. Neurotoxic side effects of taxanes on the peripheral nervous system (PNS) comprise the paclitaxel acute pain syndrome, as well as paclitaxel-induced neuropathy, the latter leading to sensory alterations such as tingling, allodynia, sensory loss, or chronic neuropathic pain [2, 4]. While taxanes, as microtubule-stabilizing drugs, lead to mitotic spindle arrest and subsequent apoptosis in dividing cancer cells, defining the molecular mechanisms leading to dysfunction and neurodegeneration in post-mitotic neuronal cells is more challenging. Previous in-vitro studies reported impaired mitochondrial trafficking, increased reactive oxygen species [7, 8] or microtubule dysfunction [9, 10] to contribute to and exacerbate axonal degeneration, while changes in neuronal calcium homeostasis with downstream caspases activation have been reported to drive neuronal apoptosis [3, 11–14]. Functional analyses attributed altered nociception to changed neuronal excitability [15], differential sodium [16] and calcium channel expression [17], TRPV1 sensitivity [18], or enhanced neuropeptide release of CGRP or substance P upon paclitaxel exposure [19]. In-vivo studies demonstrated secondary, more complex neuro-immune interactions of satellite glial cells closely coupled to dorsal root ganglia (DRG) that contribute to neuropathic pain [20], as well as the invasion of macrophages into the DRG resulting in neuronal cell death [21, 22]. Some of the typical signs of axonal sensory neuropathy [23] could be prevented by blockade of interleukin-6, underlining the essential role of neuroinflammation [24] in CIPN. Most of this knowledge has been derived from rodent-based models that suffer from relevant interspecies differences [25], and the frequently used inbred strains lack the genetic heterogeneity of humans [26, 27]. This may partly explain the lack of translation of preventive or effective symptomatic treatments from preclinical models to the patient [28], making CIPN a still unmet medical need [14].

Induced pluripotent stem cell-derived sensory neurons (iPSC-DSN) [29] are an elegant approach to model neurotoxicity or nociception in a fully human in-vitro system [30–32]. We have recently shown that iPSC-DSN replicate relevant mechanisms of CIPN in vitro [33] while being a suitable platform for translational biomarker research [34, 35].

In the present study, we obtained iPSC from five different donors, defining commonly deregulated pathways relevant for CIPN across cell lines. We conducted RNA sequencing at a variety of time points spanning from 2 h of paclitaxel exposure to 5 days after removal of paclitaxel, thereby defining cascades from an immediate response to early neuronal recovery. Unbiased RNA sequencing was coupled with deep proteome and lipidome analyses in iPSC-DSN at incipient decline of cell viability to define relevant pathways of neurodegeneration, nociception, and neuroinflammation, and outline potentially druggable targets.

## Results

### Characterization of iPSC-DSN and effects of paclitaxel exposure on cell viability

**Fig. 1:**
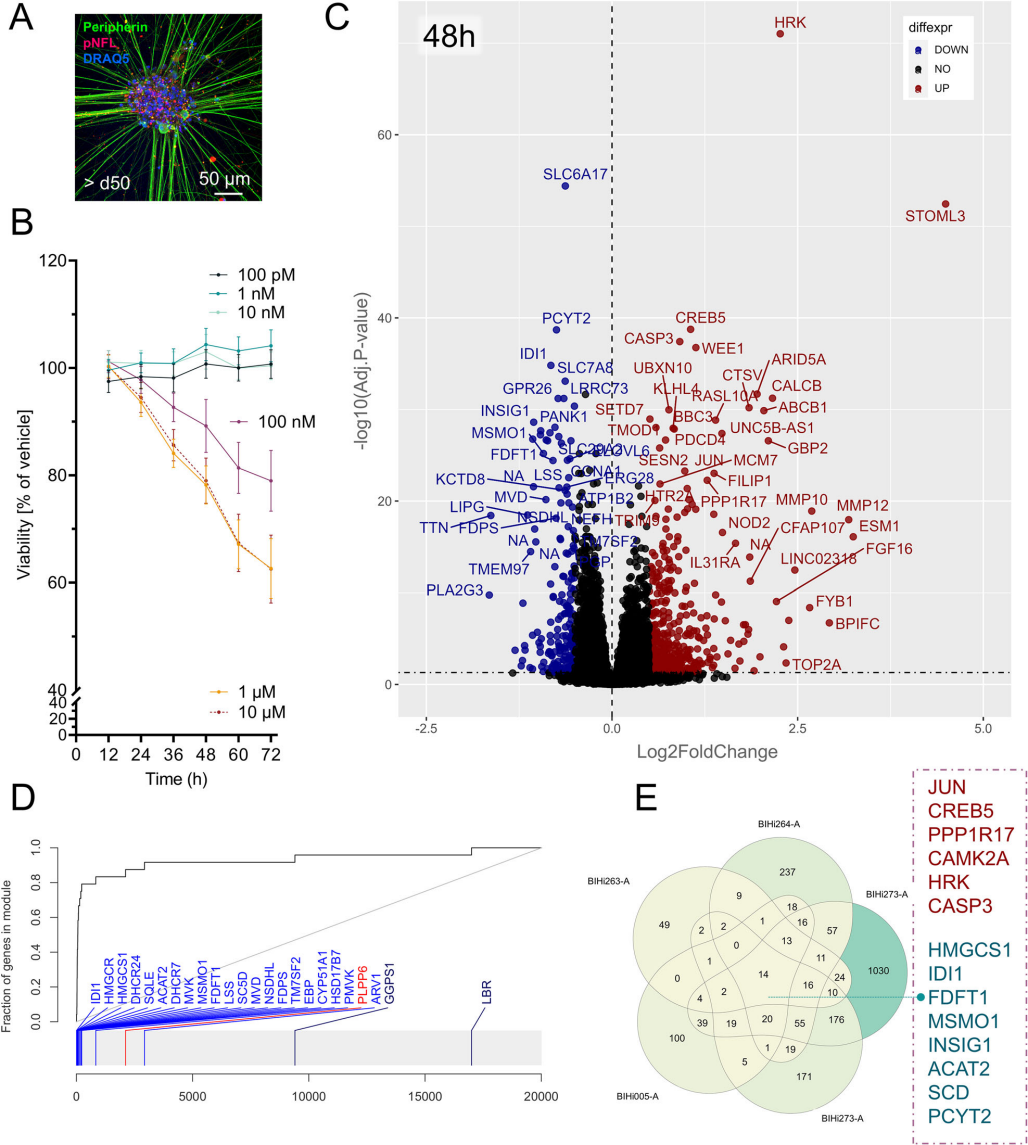
iPSC-DSN characterization and differential gene expression upon 48 h treatment with 100 nM Paclitaxel. **A** iPSC-DSN (>d50) expressed typical markers of the PNS, such as peripherin or phosphorylated neurofilament light chain (pNFL), and showed typical electrophysiological properties in patch-clamp and Ca^2+^ imaging experiments (Suppl. Fig. 1). **B** MTT-live cell assays confirmed a dose- and time-dependent decline of cell viabilities initiating at 48 h, with an IC_50_ of about 100 nM, which corresponds to the clinically applied steady state concentration (mean with 95% confidence interval). **C** At incipient decline of cell viability, i.e., 48 h after incubation with 100 nM paclitaxel, we found an overexpression of pro-apoptotic genes such as *CASP3 or PCDC4*, as well as genes of the BH3-only members of the BCL2-family *HRK*, *BBC3* (*PUMA*), or *BCL2L11* (*BIM*). Genes of the neuronal stress response, such as *JUN*, *DDIT3*, ATF3, or inflammatory genes such as *MMP10*, *MMP12*, or *IL31RA*, were found upregulated, as well as the nociception and neuronal inflammation mediating neuropeptide gene *CALCB*, the TRPV1-sensitizer *PIRT* or the PIEZO2 modulator *STOML3*, and different TNFα pathway receptors. Lipid biosynthesis-associated enzymes were markedly downregulated (Table 1). **D** Evidence plots confirmed a downregulation of almost all genes relevant for *Cholesterol Biosynthesis*. **E** Venn-Diagram revealed an overlap of 14 genes that were significantly deregulated across all cell lines, including the aforementioned genes of neuronal stress response and apoptosis, *JUN*, *HRK*, or *CASP3*, the calcium calmodulin kinase *CAMK2A* and lipid biosynthesis enzymes. There was a high correlation of the differentially expressed genes across different cell lines (Suppl. Figs. 2B, C and 3). *1* *A:* Representative staining of BIHi264-A. **B** Pooled results of 5 iPSC-DSN cell lines (BIHi263-A, BIHi264-A, BIHi272-A, BIHi273-A, BIHi005-A) with 6 technical replicates per condition per cell line. **C**–**E** Pooled experiments of 30 replicates (wells) from 5 experimental units (plates) of 5 genetically distinct iPSC-DSN cell lines.

To investigate mechanisms of neurotoxicity in vitro, iPSC-DSN were generated from five genetically distinct stem cell donors, s.c. BIHi263-A, BIHi264-A, BIHi272-A, BIHi273-A (all female paclitaxel-treated breast-cancer patients, two with CIPN, two without CIPN [36]) and the reference cell line BIHi005-A (male, healthy, [33]), see *Methods* and Suppl. Table 1. Sensory neurons (assessed >d50) expressed typical markers of the (peripheral) neuro-axonal cytoskeleton such as peripherin and neurofilament light chain, NFL (Fig. 1A). Morphological properties, typical sensory neuron marker expression, detailed electrophysiological characterization, and calcium imaging responses of iPSC-DSN are shown in Suppl. Figure 1A–H and in [33, 37]. The application of paclitaxel led to a time and dose-dependent decline of cell viability in all investigated cell lines: We found that paclitaxel (PTX) concentrations of 0.1–10 nM did not substantially affect cell viability even after prolonged incubation. A clinically relevant paclitaxel dose of 100 nM led to a decrease of cell viability commencing at 48 h (viability: 89 ± 12%). As this marks the onset of toxic neurodegeneration, both the time point and dose were selected for later integrated transcriptomic, proteomic, and lipidomic analyses. Supratherapeutic doses of paclitaxel between 1 and 10 µM further decreased cell viability (paclitaxel 1 µM at 72 h: 63 ± 13%, Fig. 1B). At 100 nM, viabilities plateaued after 84 h (viability: 76 ± 4%), whereas supratherapeutic concentrations continued to reduce viability even beyond 108 h (Suppl. Fig. 2A).

### iPSC-DSN transcriptome at incipient paclitaxel-induced neurotoxicity

The clinically relevant dose of 100 nM paclitaxel at 48 h led to a marked upregulation of typical genes of the neuronal stress response and injury, such as *JUN, WEE1, ARID5A, SESN2*, *ATF3*, *GADD45A*, intrinsic apoptotic pathways such as HRK, *BBC3 (PUMA), BCL2L11 (BIM)* or the effector caspase *CASP3* (Fig. 1C and Table 1*)*. iPSC-DSN overexpressed signatures of neuroinflammation and nociception with an upregulation of the nociceptor neuropeptide *CALCB*, extracellular matrix remodeling enzymes such as *MMP10, MMP12*, interleukin receptors such as *IL31RA*, and the mechanosensory modulator *STOML3* (Fig. 1C and Table 1). Down-regulated genes comprise key enzymes of lipid biosynthesis such as *LSS*, *HMGCS1*, *HMGCR, or IDI1* (Fig. 1C, D and Table 1). Analyzing the transcriptome of the five iPSC-DSN cell lines separately revealed a consistent deregulation of these genes across cell lines (Fig. 1E and Suppl. Fig. 2B for concordance-discordance [DISCO] plots, Suppl. Fig. 3 for co-expression analyses, Suppl. Table 2 for all differentially expressed genes).

**Table 1:** Differentially expressed genes in iPSC-DSN treated with 100 nM paclitaxel vs DMSO in the course of time and their relevance.

Symbol/alias	Log2FC^48h^	Relevance
		**Early response (12–24** **h)**
*JUN*	+0.98	Transcription factor, subunit of AP-1, induced by various extracellular stressors, neuronal injury [38]; involved in proliferation, transformation, cell death [39]. Apoptosis regulation in neurons via the intrinsic (mitochondrial) apoptotic pathway, activating pro-apoptotic BH3-only family members such as HRK, PUMA (BBC3) or BIM (BCL2L11), leading to mitochondrial outer membrane permeabilization and CASP3 activation [41]. Activation in dorsal root ganglia associated with neuroinflammation and neuropathic pain in animals [113, 114] and humans [25]; involved in axonal degeneration by upregulation of puma in sensory neuron somata [47]; upregulation drives inflammation and neurodegeneration in models of Alzheimer’s disease [115, 116] and amyotrophic lateral sclerosis (ALS) [117]; upregulated from 12h-168h and on protein level.
*SH2D3C/ NSP3*	+0.74	Adapter protein, involved in cell adhesion and migration, integrin pathway; overexpression accelerates cell death in models of Alzheimer’s disease (AD) [118], upregulated from 12h-168h
		**Intermediate response (24–48** **h)**
		Genes of neuronal stress response, apoptosis, neurodegeneration
*HRK*	+2.3	Activator of intrinsic apoptosis by the selective interaction with anti-apoptotic Bcl-2 family members via its BH3-domain (s.c. BH3-only protein family member), upregulated upon, e.g., axonal injury by c-JUN [41, 43]; role in neuronal apoptosis debated [119]
*BBC3/ PUMA*	+0.84	Pro-apoptotic BH3-only protein, inhibitor of anti-apoptotic proteins; direct activator BAX/BAK inducing mitochondrial outer membrane permeabilization and apoptosis [41, 44, 47], activated upon nerve injury in neuronal somata to coordinate downstream axon degeneration [47]; involved in amyloid beta toxicity in AD [116] and motor neuron disease [120]
*BCL2L11/BIM*	+0.69	Pro-apoptotic BCL2-protein family member (BH3-only protein member); enables BAX activation and intrinsic neuronal apoptosis [41, 121]
*PDCD4*	+0.72	Translation repressor, promotes activation of microglia, facilitates neuronal apoptosis during neuroinflammation [122]
*CASP3*	+0.91	Central effector caspase of intrinsic and extrinsic apoptotic pathways [41]
*ATF3*	+0.89	Transcription factor, rapidly induced after neuronal injury, involved in axonal regeneration [123], neuropathic pain [124], CIPN [33, 125, 126]
*DDIT3/CHOP*	+0.55	Transcription factor of the endoplasmatic reticulum stress response; induced by, e.g., JUN activation [118]; positive regulation of inflammation (e.g., IL6, IL8, TNFα receptors); induction of pro-cell death signals (e.g., BBC3/PUMA); JUN/DDIT3 as key molecular hubs of neuronal apoptosis upon axonal injury [127]
*DUSP16*	+1.03	Phosphatase; inactivates mitogen-activated-protein kinases by dephosphorylation, specifically members of the JNK and ERK-pathway family; negative regulation of the pro-degenerative factor puma (BBC3); preserving factor against axonal degeneration [128]
*CREB5*	+1.06	Transcription factor, homo- or heterodimerizes with c-JUN; mediates tissue regeneration and plasticity after injury [129, 130]
*GADD45A*	+0.69	DNA repair gene [131]; correlated with sensory neuron survival upon injury [131]
		*Other relevant members in this group:* e.g., *CX3CR1* [−1.17]; *EIF2AK3* [+0.88]; *PPP1R17* [+1.28]; *SESN2* [+0.64];
		*WEE1* [+1.13]
		Neuroinflammation, nociception, signal transduction associated genes
*CALCB*	+2.16	Neuropeptide in sensory neurons, implicated in neurogenic inflammation, vasodilatation; facilitator of nociceptive transmission (peripherally and centrally) [132], involved in CIPN [133], migraine pain [134]
*PIRT*	+0.75	Regulatory subunit of TRPV1; expressed in nociceptive neurons, potentiates responsiveness to noxious heat [135]; transcriptional changes in PIRT^+^ neurons involved in the pathogenesis of neuropathic pain [136]
*MMP10*	+2.69	Peptidase, involved in degradation of extracellular matrix in physiological states and inflammation; overexpression associated with neuroinflammation, neuronal senescence and neurodegeneration [137, 138]
*ARID5A*	+1.95	RNA binding protein; selective stabilizer of mRNA of, e.g., IL6; promoter of neuroinflammation [139]
*IL31RA*	+1.67	Cytokine receptor expressed in monocytes and sensory neurons, involved in neuropathic itch [64, 140]
*IL6R*	+0.55	Cytokine receptor, upregulated in sensory neurons upon, e.g., bortezomib or paclitaxel treatment, inhibition of the IL-6 pathway leads to mitigation of CIPN [24, 141]
*CYSLTR2*	+1.84	Leukotriene receptor with high expression in rodent and human DRG; activation mediates acute and chronic itch [65], microglial inflammation and neurotoxicity [142]
*C3AR1*	+1.77	Complement receptor, involved in paclitaxel induced neurotoxicity and pain in rats [68], microglial activation [143]
*ICAM1*	+0.99	Cell surface glycoprotein of endothelial cells and neurons facilitating leukocyte infiltration, inflammation and pain in CIPN [67]
*EGR1*	+1.10	Transcriptional regulator, expressed after sensory neuron injury and involved in the development of neuropathic pain [124]
*STOML3*	+4.49	Potent modulator of mechanotransduction in sensory neurons by interaction with PIEZO2 [144]; involved in mechanical hypersensitivity [74]; markedly upregulated from 24-168 h
*CAMK2A*	+0.88	Serine/threonine protein kinase and transcription regulator, expressed in nociceptors, involved in tissue injury evoked pain [73]
		*Other relevant members in this group: e.g., BDKRB2* [+0.87], *GABRQ* [−0.70], *GABRR1* [−0.61], *GALR1* [−0.83], *GBP2* [+2.1]; *GRIN3A* [−0.69], *HTR2A* [+1.01], *KCNK12* [+0.77], *MRGPRX4* [+1.10], *MMP12* [+3.19]; *NPFFR2* [+1.01]; *NDRG1* [+0.89]; *PROKR1* [+0.83], *TNFAIP8* [−0.48], *TNFRSF12A* [+0.61], *TRPV3* [+0.83]—see Fig. 2.
		Lipid metabolism
*LSS*	−0.71	First step of cholesterol synthesis
		*Other relevant members in this group: e.g., DHCR24* [−0.83]; *HMGCS1* [−0.95]; *HMGCR* [−0.77]; *IDI1* [−0.82]; *MSMO1* [−0.92]; *TMEM97* [−1.10]; *SQLE* [−0.72]
		**Late response (72–168** **h)**
*FGF16*	+2.21	Growth factor, FGF members released from degenerating neurons induce microglia invasion and phagocytosis of debris [75]
*ABCB1*	+2.04	Multidrug resistance transporter family member; efflux of drugs such as paclitaxel across the membrane; genetic polymorphisms associated with CIPN [76].

Log2FC refers to differential expression of all 5 iPSC-DSN cell lines pooled upon 48 h treatment with 100 nM paclitaxel vs DMSO.

General information for the respective genes was derived from the genecards database [145].

*ABCB1* ATP Binding Cassette Subfamily B Member 1, *ARID5A* AT-Rich Interaction Domain 5A, *ATF3* activating transcription factor 3, *BBC3/PUMA* BCL2 binding component 3, *BCL2L11/BIM* BCL2 Like 11, *BDKRB2* Bradykinin Receptor B2, *C3AR1* complement C3a receptor 1, *CALCB* calcitonin related polypeptide beta, *CAMK2A* calcium/calmodulin dependent protein kinase II alpha, *CASP3* caspase 3, *CREB5* CAMP responsive element binding protein 5, *CX3CR1* C-X3-C motif chemokine receptor 1, *CYSLTR2* cysteinyl leukotriene receptor 2, *DDIT3/CHOP* DNA damage inducible transcript 3, *DHCR24* 24-dehydrocholesterol reductase, *DUSP16* dual specificity phosphatase 16, *EGR1* early growth response, *FGF16* fibroblast growth factor 16, *GABRQ* gamma-aminobutyric acid type A receptor theta subunit, *GADD45A* growth arrest and DNA damage inducible alpha, *GABRR1* gamma-aminobutyric acid type A receptor Rho1 subunit, *GALR1* galanin receptor 1, *GBP2* guanylate binding protein 2, *GRIN3A* glutamate ionotropic receptor NMDA type subunit 3A, *EIF2AK3* eukaryotic translation initiation factor 2 alpha kinase 3, *HMGCR* 3-hydroxy-3-methylglutaryl-CoA reductase, *HMGCS1* 3-hydroxy-3-methylglutaryl-CoA synthase, *HRK* activator of apoptosis harakiri, *HTR2A* 5-hydroxytryptamine (serotonin) receptor 2A, *IDI1* isopentenyl-diphosphate delta isomerase 1, *ICAM1* intercellular adhesion molecule 1, *IL6R* interleukin 6 receptor, *IL31RA* interleukin 31 receptor A, *JUN* jun proto-oncogene, AP-1 transcription factor subunit, *KCNK12* potassium two pore domain channel subfamily K member 12, *LSS* lanosterol synthase, *MMP10* matrix metallopeptidase 10, *MSMO1* methylsterol monooxygenase 1, *MMP12* matrix metallopeptidase 12, *MRGPRX4* MAS related GPR family member X4, *NDRG1* *N*-Myc downstream regulated gene 1, *NPFFR2* neuropeptide FF receptor 2, *PDCD4* programmed cell death 4, *PIRT* phosphoinositide interacting regulator of transient receptor potential channels, *PPP1R17* protein phosphatase 1 regulatory subunit 17, *PROKR1* prokineticin receptor 1, *SH2D3C/NSP3* SH2 domain containing 3C, *SESN2* sestrin 2, *STOML3* stomatin like 3, *SQLE* squalene epoxidase, *TNFAIP8* TNF alpha induced protein 8, *TNFRSF12A/FN14* TNF receptor superfamily member 12A/fibroblast growth factor-inducible immediate-early response protein 14, *TMEM97* transmembrane protein 97, *TRPV3* transient receptor potential cation channel subfamily V member 3, *WEE1* WEE1 G2 checkpoint kinase.

**Fig. 2:**
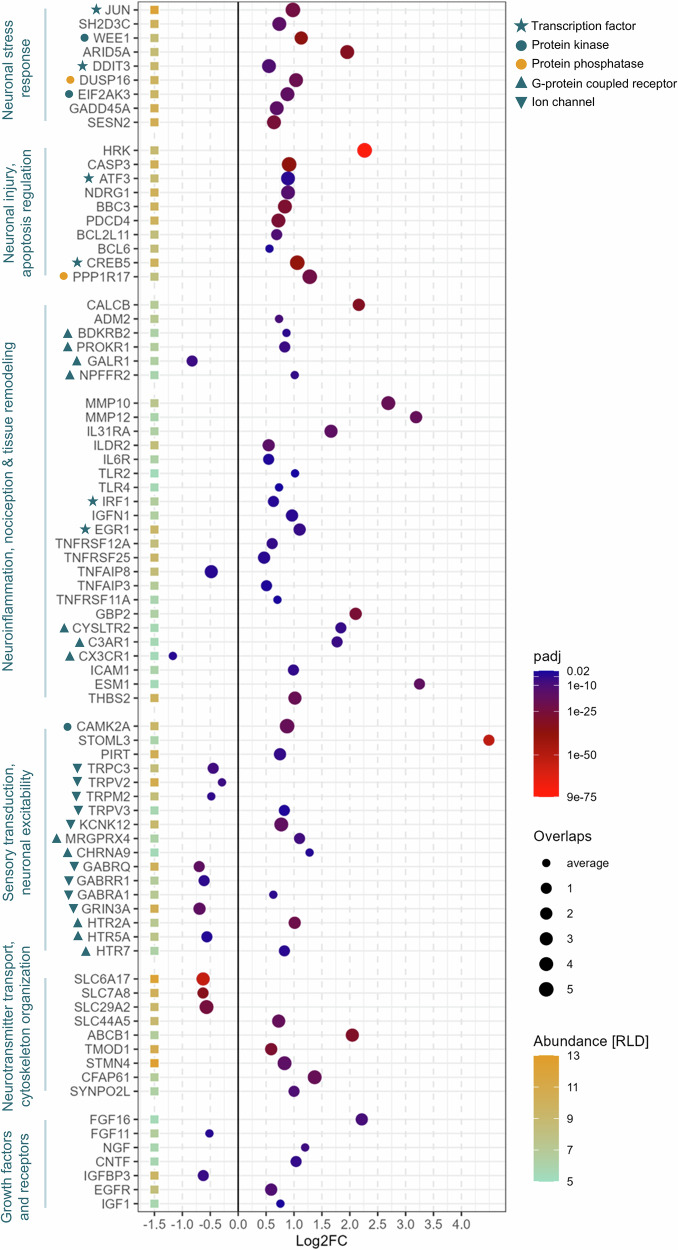
Transcriptomic signatures relevant to the pathophysiology of chemotherapy-induced peripheral neuropathy (CIPN). Differentially expressed genes were functionally grouped based on their mechanistic contribution to the CIPN phenotype. These included: transcription factors (*JUN*, *ATF3*, *CREB5*), protein kinases (*WEE1*, *CAMK2A)*, and g-protein coupled receptors involved in nociceptive signaling, such as bradykinin, prokineticin, galanin, and neuropeptide receptors (*BDKRB2*, *PROKR1*, *GALR1*, *NPFFR2*). Neuroinflammatory signatures encompassed genes associated with tissue remodeling (*MMP10*, *MMP12*, *ICAM1*), cytokine and immune receptors for interleukins (*IL31RA*, *ILDR2*, *IL6R*), TNFα (*TNFRSF12A*, *TNFRSF25*), leukotrienes (*CYSLTR2*), interferon (*IRF1*), complement (*C3AR1*), and Toll-like receptors (*TLR2*, *TLR4*). We also observed differential expressions of genes involved in sensory transduction, including: vanilloid receptors (down: *TRPC3*, *TRPV2*, *TRPM2*; up: *TRPV3*), potassium channels (*KCNK12*), neurotransmitter receptors for acetylcholine (*CHRNA9*), GABA (*GABRR1*), glutamate (*GRIN3A*), and serotonin (*HTR2A*, *HTR5A*, *HTR7*). Additional alterations were seen in neurotransmitter reuptake transporters (*SLC6A17*) and drug efflux transporters (*ABCB1*), as well as growth factors and their receptors (*FGF16*). Further, growth factors and their receptors differed substantially between paclitaxel and DMSO-treated sensory neurons. Data reflect pooled transcriptomic profiles from 30 wells across 5 experimental runs applying 5 genetically distinct iPSC-derived sensory neuron (iPSC‑DSN) lines. Genes are sorted by function and annotated by: Benjamini-Hochberg adjusted *p*-values (padj), degree of overlaps across cell lines (i.e., in how many of the 5 lines the padj was < 0.05), and Regularized Logarithm Transformation (rlog or RLD). Most consistent and biologically relevant genes are highlighted in Table 1.

In the next step, we searched the differentially expressed genes and annotated them by groups based on their function and potential relevance in the context of chemotherapy induced peripheral neuropathy (Fig. 2). We identified several transcription factors (e.g., *JUN, DDIT3, ATF3, CREB5, EGR1*), protein kinases (*WEE1, EIF2AK3, CAMK2A*), phosphatases (*DUSP16, PPP1R17*), g-protein coupled receptors modulating nociception (*BDKRB2, PROKR1, GALR1, MRGPRX4, HTR2A*) or neuroinflammation (*CYSLTR2, C3AR1, CX3CR1*), as well as ion channels (*TRPV3, KCNK12, GABRQ, GABRR1, GRIN3A*) that are involved in sensory transduction and neuronal excitability. Finally, we found that paclitaxel led to a differential expression of transporter genes (*SLC6A17, SLC29A2, ABCB1*), cytoskeleton elements (*STMN4*), and growth factors and receptors (*FGF16, EGFR, CNTF;* Table 1).

### Temporal transcriptomic profiling of toxic neurodegeneration

**Fig. 3:**
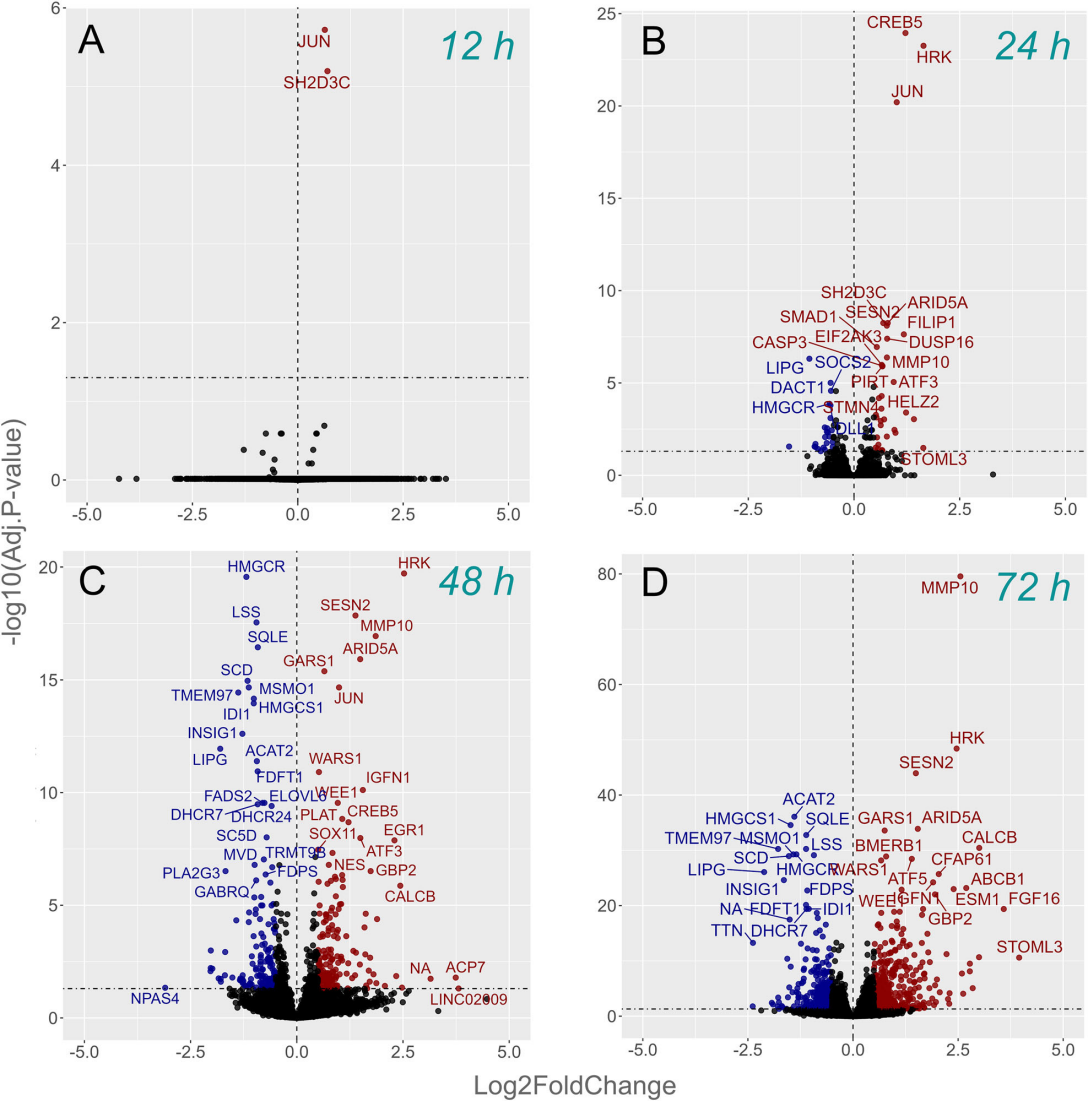
Temporal transcriptomic signatures of paclitaxel-induced neurotoxicity. The number of differentially expressed genes progressively increased with the duration of paclitaxel exposure (red: upregulated; blue: downregulated). While no significantly deregulated genes were found at 2 h or 6 h (Suppl. Fig. 5A, B), the neuronal stress gene *JUN* appeared differentially expressed as early as 12 h after paclitaxel exposure (**A**). At 24 h (**B**), first neuronal injury and apoptosis associated genes such as *CASP3*, *ATF3*, *HRK* or *BBC3* appeared, as well as genes restricting JNK-pathway activation such as *DUSP16*, inflammation and nociception associated genes such as *MMP10* or *CALCB* that plateaued at 48 h of paclitaxel exposure (**C**), while lipid biosynthesis-associated genes appeared downregulated (e.g., *HMGCR*). These transcriptomic signatures were preserved, even 24 h (**D**) and 5 d after removal of the drug (168 h timepoint, Suppl. Fig. 5C). The differential expression of lipid biosynthesis associated genes, as well as the drug efflux transporter *ABCB1* and the growth factor *FGF16* further increased in magnitude after removal of the drug (Suppl. Fig. 5C). Data represent 42 biological replicates (wells) from iPSC-derived sensory neurons (iPSC‑DSN BIHi264‑A) across 7 experimental plates (1 per timepoint), with matched paclitaxel- and DMSO-treated wells (*n* = 3 per condition per timepoint).

To approach cascades of neurotoxicity, RNA sequencing was conducted at multiple timepoints of 100 nM paclitaxel exposure, i.e., at 2, 6, 12, 24 and 48 h. Further, media was washed out 48 h after paclitaxel incubation and RNA collected after additional 24 h and 5 days (72 h and 168 h timepoints). Differentially expressed genes are summarized as *early* (≤12 h), *intermediate* (24–48 h) and *late* (>72 h) response genes and set into context with their functional relevance in Table 1. At 6 h, gene enrichment analyses revealed a transient deregulation of mitochondrial gene sets (Suppl. Fig. 4). Early immediate genes were detected as early as 12 h, including *JUN* as cellular stress gene of the AP1-transcription factor network and the cell adhesion-/migration associated gene *SH2D3C* (Fig. 3A and Suppl. Figs. 5 and 6). At 24 h, these early deregulated genes remained differentially expressed while more pro-apoptotic genes, such as the BH3-only member *HRK* or *CASP3* or the neuronal injury marker *ATF3*, appeared upregulated, whereas *BBC3* (*PUMA*), as an additional pro-apoptotic gene, emerged at 48 h (Fig. 3C and Suppl. Fig. 6A–F). Typical neuroinflammation and nociception associated signatures at the 24 h timepoint included *MMP10*, while *CALCB*, *IL31RA, IL6R* and *EGR1* or *DDIT3* appeared from 48 h onwards. Lipid biosynthesis-associated genes, such as *HMGCR* or *LSS*, started to be statistically significantly downregulated at 24 h and decreased over time. In this *late phase* after removal of paclitaxel, we observed a persistent upregulation of the aforementioned genes of the cellular stress response, neuronal injury, apoptosis and neuroinflammation, while the key enzymes of lipid biosynthesis further decreased in absolute log2FC. The growth factor *FGF16* and the multidrug transporter *ABCB1* further increased after paclitaxel removal (Fig. 3D, Table 1, and Suppl. Fig. 5A–F).

### Deep proteome analysis of paclitaxel-treated iPSC-DSN

**Fig. 4:**
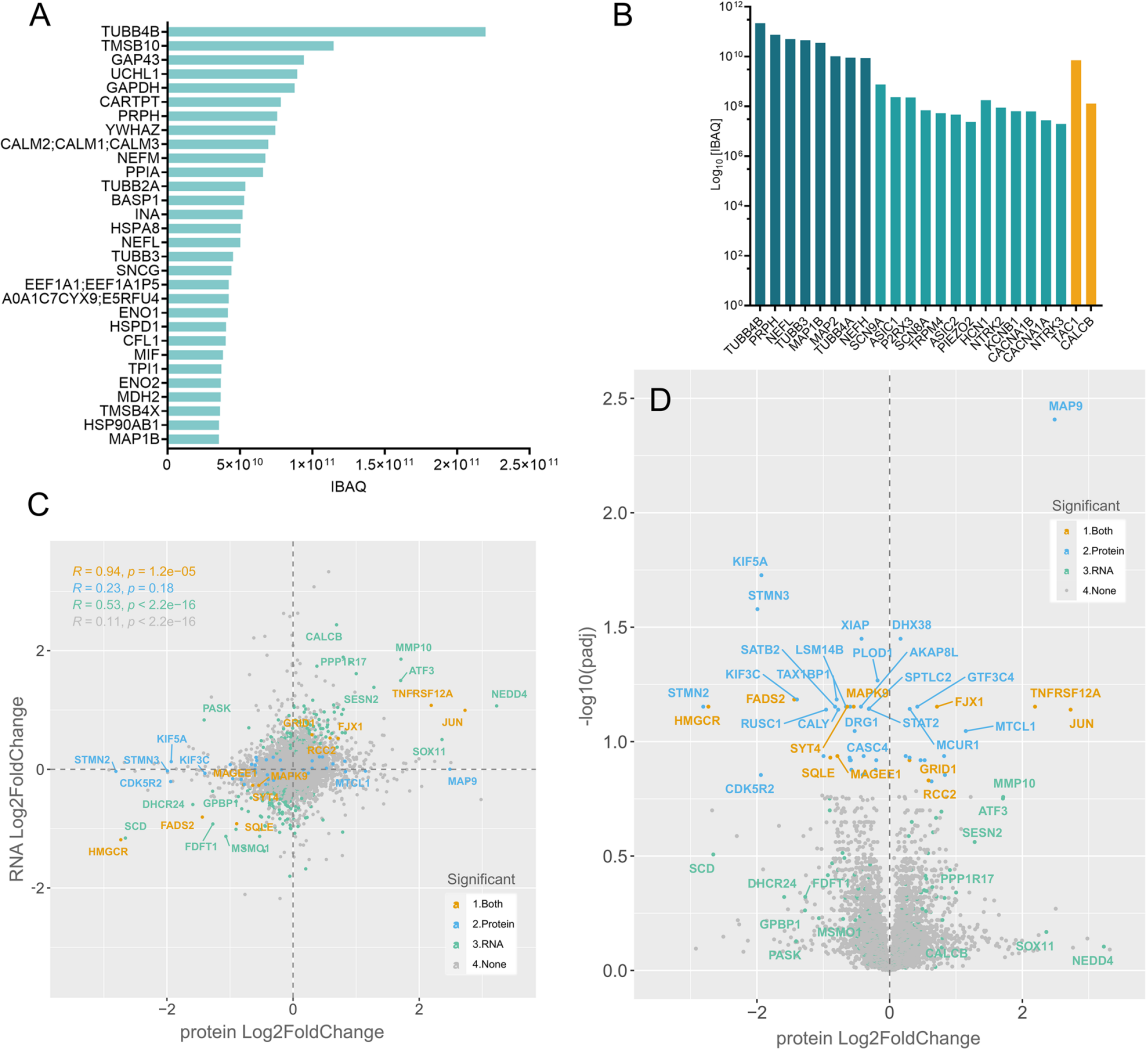
Deep proteome analyses—iPSC-DSN characterization and differentially regulated proteins upon paclitaxel exposure at 48 h of 100 nM paclitaxel exposure. **A** The 30 most abundantly expressed genes according to their IBAQ (IBAQ of proteins) comprised, besides microtubule proteins and housekeeping enzymes, typical neuronal cytoskeleton proteins such as peripherin and different neurofilaments. **B** For further characterization, we confirmed expression of typical sensory neuron-associated proteins like the vanilloid receptor (TRPV1), the sodium channel SCN9A or typical nociceptor proteins such as CALCB or tachykinin (neuropeptide y, TAC1). **C** We found a remarkable correlation between differentially expressed genes and proteins (orange), with the highest consistency for JUN, TNFRSF12A, HMGCR, and a good correlation for, e.g., kinesins and stathmins (Table 2). **D** Volcano plot of differentially expressed proteins upon 48 h 100 nM paclitaxel exposure largely confirmed transcriptomic results. Besides an approximately sixfold upregulation of JUN or fourfold upregulation of TNFRSF12A, there were marked degradations of the microtubule-associated proteins STMN2 and -3 and the organelle transport motor protein KIF5A, as well as the neuron-specific cyclin-dependent kinase 5 (CDK5R2). Colors in C-D indicate significant differential expression (adjusted *p*-value < 0.05) on both protein and RNA level (orange), only protein (blue), or only RNA (green). Proteomic analyses were performed in six samples of iPSC-DSN BIHi264-A (*n* = 3 paclitaxel- and *n* = 3 DMSO-treated wells).

For confirmatory analyses, unbiased deep proteome analyses were conducted in BIHi264-A. 8.638 proteins were identified at a peptide and protein false discovery rate (FDR) of 1%. Highest abundances according to their intensity-based absolute quantification (IBAQ) were observed for general cytoskeleton elements (e.g., TUBB4B, TMSB10, GAP4), house-keeping enzymes (UCHL1, GAPDH), as well as typical neuronal cytoskeleton elements (e.g., PRPH, NEFM, NEFL, TUBB3; Fig. 4A). Expectedly, sensory nervous system receptors were expressed such as the nociceptor receptors SCN9A (Na_V_1.7), the acid sensing ion channel ASIC1, the purinoceptor P2RX3, the mechano receptor piezo2 or the proprioceptor receptor NTRK3, as well as neuropeptides such as TAC1 (Substance P) or CALCB (Fig. 4B). Correlating RNA sequencing results with the proteome dataset, we found a modest but highly statistically significant correlation between the log2 fold changes (*R* = 0.11, *p* < 2.2 × 10^−16^) and a very strong correlation of genes with proteins that were differentially expressed in both datasets (*R* = 0.94, *p* = 1.2 × 10^−5^) (Fig. 4C). Differential proteomic analyses between 100 nM paclitaxel vs DMSO-treated iPSC-DSN (48 h), using the TMT reporter intensity signal for each sample, outlined 97 statistically significantly deregulated proteins (FDR 10%, 57 at FDR 5%) of which 38 were up- und 69 downregulated (Fig. 4D and Suppl. Table 3). These proteins are set into context of their relevance in Table 2. Consistent with RNA sequencing results, proteins of the cellular stress response, apoptosis and neurodegeneration such as JUN, ATF3, SESN2 were upregulated, as well as proteins associated with neuroinflammation or pain such as MMP10, or TNFRSF12A, while key factors of cholesterol biosynthesis such as HMGCS1, HMGCR or SCD were markedly downregulated (Fig. 4D). Notably, we found a heavy degradation of proteins involved with microtubule dynamics, axonal transport of organelles and vesicles including phosphoproteins of the neuronal cytoskeleton (STMN2 and STMN3), different motor proteins of the kinesin family (KIF5A, KIF3C) and the scaffold protein JIP3 (MAPK8IP3) that normally organize lysosome and phagosome dynamics (Fig. 4D and Table 2). Besides the aforementioned proteins, CASP3, PIRT, CALCB, DHCR24, and MSMO1 showed good RNA-protein correlations, although they did not reach the FDR 10 threshold in the proteome data set (Suppl. Tables 3 and 4).

**Table 2:** Differentially regulated proteins in iPSC-DSN treated with 100 nM paclitaxel vs DMSO at 48 h and their relevance.

Symbol/alias	Log2FC^FDR^	Relevance
		**Microtubule dynamics, axonal transport of organelles and vesicles**
STMN2, STMN3	−2.81^**^; −1.99^**^	Phosphoproteins of the (neuronal) cytoskeleton, regulators of microtubule dynamics; dysfunction associated with motor neuron disease, Alzheimer disease; STMN2 loss drives axonal collapse leading to motor and sensory denervation [82–85]
KIF5A	−1.93^**^	Kinesin, microtubule motor of intracellular organelle transport; e.g., axonal transport of mitochondria [146, 147], loss of or decreased function associated with neurodegeneration in spastic paraplegia (HSP10), Charcot-Marie-Tooth Disease Typ 2 (CMT2), familial amyotrophic lateral sclerosis [79–81, 148]
KIF3C	−1.40^**^	Kinesin, controls microtubule dynamics in the growth cone, required for axon growth and regeneration [149]
JIP3/MAPK8IP2	−1.19^**^	Neuronal scaffold protein, interacts with dynein and kinesin 1 to regulate bidirectional organelle transport [150], lysosome clearance; mutations associated with focal accumulations of lysosomes and axonal swellings in neurodegeneration, e.g., in Alzheimer’s disease [151–153]
RETREG1/FAM134B	−0.98^**^	Endoplasmatic reticulum (ER) anchored autophagy regulator and receptor (Golgi protein); downregulation associated with ER expansion, inhibition of ER turnover and sensitization to stress-induced apoptosis [154]; necessary for long-term survival of nociceptors, autonomic ganglia; mutation causes hereditary sensory and autonomic neuropathy type II (HSAN II) [155, 156]
		*Other relevant members in this group:* e.g., CALY [−0.78^**^]; GBF1 [−0.66^*^] [157]; JIP4/SPAG9 [−2.68^**^]; KIF1B [−0.78^*^] [158]; KIF26B [−0.69^*^] [159]; MAP9 [−1.1^**^] [160]; MTCL1 [+1.15^**^]; MTMR4 [−1.91^*^]; MTMR6 [−0.73^*^]; PCM1 [−0.94^**^] [161]; SYT4 [−0.64^*^]; TAX1BP1 [−0.72^*^] [162]; TUBA4A [+1.01^*^] [163]; TUBB4A [+0.82^**^]
		**Proteins of neuronal stress response, apoptosis, neurodegeneration**
JUN	+2.72^**^	see Table 1
ATF3	+1.71^**^	see Table 1
SESN2	+1.28^**^	Regulator of cell growth and survival; downregulates MTORC1 signaling, restoration of autophagy, suppresses neuro-inflammation [164]; antioxidant function, limits neuropathic pain [165].
CDK5R2	−1.94^**^	Neuron specific activator of CDK5 kinase; overactivation associated with neuroinflammation in neurodegeneration [166] and inflammatory pain [167]; mitigation of CDK5 activation neuroprotective in spinal muscular atrophy [168]
		*Other relevant members in this group:* e.g., CDKN2D [−0.62^*^]; DNAJB9 [−0.71^**^]; MAPK9/JNK2 [−0.54^*^]; STAT3 [−0.84^**^]; VGLL4 [+1.22^**^] [169]
		**Neuroinflammation and pain associated proteins**
TNFRSF12A/FN14	+2.19^**^	TWEAKR (TNF-related weak inducer of apoptosis receptor); overexpressed upon neuronal injury [170];
		induces apoptosis via the extrinsic apoptotic pathway [171]; involved in neuropathic pain via NF-κB activation in primary sensory neurons [172]; disturbs synaptic transmission [170]
MMP10	+1.71^**^	see Table 1
		**Lipid metabolism**
HMGCR	−2.73^**^	Rate limiting enzyme for cholesterol synthesis; role in neuropathies and neurodegeneration controversial [173–175]
HMGCS1	−0.91^**^	Enzyme that catalyzes the HMG-CoA synthesis; essential for maintaining neuronal cell membrane integrity; massive downregulation found in spinocerebellar ataxia 2 (SCA2) [176], upregulated in peripheral nerve injury [177]
SCD	−2.66^**^	Enzyme involved in fatty acid synthesis, primarily oleic acid; dysregulation in diabetic polyneuropathy [178]
		*Other relevant members in this group:* e.g., DGKD [−0.75^*^]; FADS2 [−1.44^**^]; FAR1 [−0.79^*^]; SQLE [−0.89^**^]

Numbers in brackets indicate Log2FC on protein level (paclitaxel vs DMSO).

General information for the respective proteins was derived from the genecards database [145].

*ATF3* activating transcription factor 3, *CALY* calcyon neuron specific vesicular protein, *CDKN2D* cyclin dependent kinase inhibitor 2D, *CDK5R2* cyclin dependent kinase 5 regulatory subunit 2, *DGKD* diacylglycerol kinase delta, *DNAJB9* DnaJ heat shock protein family (Hsp40) member B9, *GBF1* Golgi Brefeldin A resistant guanine nucleotide exchange, *FADS2* fatty acid desaturase 2, *FAR1* fatty acyl-CoA reductase 2, *HMGCR* 3-hydroxy-3-methylglutaryl-CoA reductase, *HMGCS1* HMG-CoA synthase isoenzymes 1, *JIP3/MAPK8IP2* C-Jun-amino-terminal kinase-interacting protein 2/mitogen-activated protein kinase 8 interacting protein 3, *JIP4/SPAG9* C-Jun-amino-terminal kinase-interacting protein 4, mitogen-activated protein kinase 8-interacting protein 4, *JUN* Jun proto-oncogene, AP-1 transcription factor subunit, *KIF1B* kinesin family member 1B, *KIF26B* kinesin family member 26B, *KIF3C* kinesin heavy chain isoform 3C, *KIF5A* kinesin heavy chain isoform 5A, *MAP9* microtubule associated protein 9, *MAPK9* mitogen-activated protein kinase, *MMP10* matrix metallopeptidase 10, *MTMR4/6* myotubularin-related protein 4/6, *MTCL1* microtubule crosslinking factor 1, *PCM1* pericentriolar material 1, *RETREG1/FAM134B* reticulophagy regulator 1, *SESN2* sestrin 2, *SCD* stearoyl-CoA desaturase, *SQLE* squalene epoxidase, *STMN2* stathmin 2, *STMN3* stathmin 3, *STAT3* signal transducer and activator of transcription, *SYT4* synaptotagmin-4, *TAX1BP1* Tax1 binding protein 1, *TNFRSF12A/FN14* TNF receptor superfamily member 12A/fibroblast growth factor-inducible immediate-early response protein 14, *TUBA4A* tubulin alpha 4a, *TUBB4A* tubulin beta 4A class IVa, *VGLL4* vestigial like family member 4.

^**^Indicates FDR5.

^*^FDR10.

### Effects of paclitaxel exposure on lipid metabolism

**Fig. 5:**
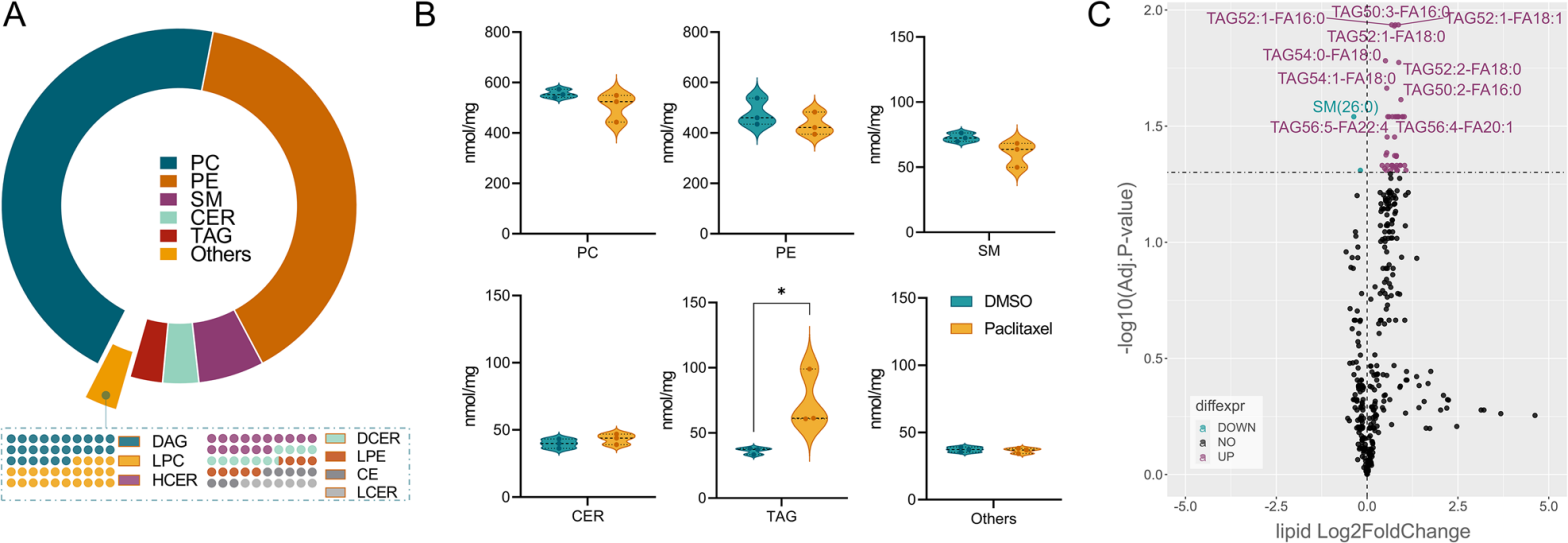
Lipidomic characterization of iPSC-DSN and effects of paclitaxel exposure on sensory neuron lipidome. Targeted lipidome analyses revealed high expression of phosphatidylcholines (PC) and phosphatidylethanolamines (PE) in iPSC-DSN, followed by sphingomyelins (SM, **A**). Paclitaxel exposure of 100 nM for 48 h leads to a trend towards a downregulation of PC, PE, and SM, but a significant upregulation of triacylglycerides (TAG, **B**). **C** Volcano plot showed mainly an upregulation of members of TAG, while only SM(26:0) and the diacylglycerol DAG(12:0/18:1) were found to be significantly downregulated on lipid level. A-C: 12 replicates (wells) of iPSC-DSN were included (all BIHi264-A), with 6 paclitaxel vs 6 DMSO-treated wells (2 wells pooled for 1 analysis). B: Unpaired *t*-test, *p* = *0.0426*. PC, phosphatidylcholine; PE, phosphatidylethanolamine; SM, sphingomyelin; CER, ceramide; TAG, triacylglycerol.

As we observed marked changes in our transcriptome and proteome analyses with regards to lipid biosynthesis after paclitaxel exposure, for confirmation, we characterized the lipid composition in iPSC-DSN of BIHi264-A. We explored whether the differential expression of lipid biosynthesis’ key enzymes influenced neuronal lipid composition: iPSC-DSN consisted of the main lipid classes phosphatidylcholine (PC, 46%), phosphatidylethanolamine (PE, 39%), sphingomyelin (SM, 6%), ceramides (CER, 3%), triacylglycerol (TAG, 3%), and others (3%) (Fig. 5A). Upon 48 h paclitaxel exposure, principal component analyses (PCA) revealed a clear difference between paclitaxel- and vehicle-treated samples (Suppl. Fig. 7). There was a trend towards a downregulation of the membrane lipids PC, PE and SM, while TAGs were significantly upregulated (Fig. 5B). We detected 412 lipids in iPSC-DSN, out of which 44 were found to be differentially expressed between vehicle and paclitaxel treatment, with 42 upregulated (all belonged to TAG) and 2 downregulated lipids (SM[26:0] and PC[16:0/20:1]) (Fig. 5C).

## Discussion

In this study, we performed an in-depth analysis of mechanisms underlying the development of paclitaxel-induced neurotoxicity in the course of time, applying a fully human in-vitro model of iPSC-DSN. Our main findings are: first, in line with previous reports [33, 34], treatment of iPSC-DSN with paclitaxel at a clinically relevant dose of 100 nM robustly decreases cell viability between 48 h and 72 h in all five investigated cell lines. Second, we found early transcriptomic signatures of neuronal stress response, including *JUN*, which were followed by transcriptional signatures of apoptosis, neuroinflammation, and nociception, whereas key enzymes of lipid biosynthesis were reproducibly downregulated. Functionally, many of these genes belong to transcription factors, kinases, phosphatases, g-protein coupled receptors, or ion channels that modulate neuronal excitability downstream. Most of these signatures were consistently found across cell lines. Third, altered proteomic and lipidomic signatures corroborated with deregulated pathways from transcriptomic data and highlighted a marked degradation of kinesins and scaffold proteins, which are essential for axonal transport and microtubule dynamics.

We investigated the temporal dynamics of transcriptional changes as early as 2 h after paclitaxel exposure until incipient neurodegeneration and recovery. In the *early phase* of paclitaxel exposure, we found evidence for deregulated mitochondrial gene sets as early as 6 h after paclitaxel exposure which were followed by a persistent upregulation of *JUN* starting at 12 h. *JUN* is a transcription factor and part of the AP-1 network which is inducible by various extracellular stressors or neuronal injury that may finally orchestrate apoptosis and neuroinflammation downstream [38–40]. *JUN* is activated in neurons by JNK3 mediated phosphorylation followed by its translocation into the nucleus inducing the transcription of pro-apoptotic BH3-only proteins [41, 42]. To this end, we found a marked and persistent upregulation of the pro-apoptotic BCL2-family members *HRK*, *BBC3 (PUMA)*, *BCL2L11 (BIM)*, as well as the effector caspase *CASP3* in the *intermediate phase* of paclitaxel exposure. These pro-cell death signals either inhibit anti-apoptotic proteins interacting with their BH3-domain (BH3-only members, e.g., *HRK* [43], *BBC3* [44, 45]) or directly activate BAX/BAK (e.g., *BBC3*, *BIM* [44, 45]) for mitochondrial outer membrane permeabilization inducing the neuronal intrinsic apoptotic pathway [41, 46] or axonal degeneration [47]. Inducers of the *extrinsic* pathway included the upregulated pro-apoptotic membrane located TNFα receptor *TNFRSF12A* (Fn14, TNF-like weak inducer of apoptosis receptor) [48, 49], as well as *TNFRSF25* (DR3, death receptor 3), while the anti-apoptotic *TNFAIP8* [50] was downregulated. This suggests a shift towards a pro-apoptotic milieu in the intermediate phase of paclitaxel exposure that timely coincides with the decrease of cell viabilities, and may, at least in part, explain neuroaxonal degeneration of sensory nerves we observe in patients [14, 34]. In parallel, molecular signatures of neuroinflammation and nociception emerged in the iPSC-DSN model that remarkably overlap with animal based literature [25, 51] or human DRG [52]. These included both neuropeptides directly mediating nociception, as well as cytokines and cytokine receptors that perpetuate neuro-inflammation or attract leukocytes: The neuropeptide *CALCB* was markedly upregulated from 24 h onwards, which facilitates firing in nociceptors and is associated with peripheral and central pain sensitization [53], as well as increased release in rodent in-vitro models of CIPN [54, 55], and may also be involved in the paclitaxel acute pain syndrome [56]. This early but persistent upregulation also held true for *PIRT*, a typical TRPV1 sensitizer [55], or the endopeptidases *MMP10 and MMP12*, which are involved in tissue remodeling and the regulation of neuropathic pain [57–59]. Paclitaxel induced a shift towards pro-inflammatory cytokine receptor expression on iPSC-DSN with the upregulation of TNFα (*TNFRSF12A, TNFRSF25*), interleukin (*IL31RA*), leukotriene (*CYSLTR2*), complement receptors (*C3AR1*) and *IL6R* as established neuroinflammatory drivers in the pathogenesis of different polyneuropathies [14, 24, 60–63] or neuropathic itch [64, 65]. Intriguingly, several preclinical trials demonstrate a pivotal, potentially druggable, role of pro-inflammatory cytokines in the pathogenesis of paclitaxel-induced polyneuropathy [24, 66]. We identified the differential expression of genes that favor the adhesion and transmigration of leukocytes (e.g., *ICAM1* [67]), microglial activators (*C3AR1* [68], *CX3CR1* [69], *GBP2* [70]), as well as transcription factors perpetuating proinflammatory cytokines downstream such as *IRF1* [71] or *EGR1* [72]. Remarkably, these neuroinflammatory signatures expected to evolve in complex tissue environments emerged within a reductionist, nearly pure iPSC-DSN system, underscoring the intrinsic capacity of sensory neurons to initiate neuro-immune interactions upon paclitaxel exposure. Regarding modulators of sensory transduction, we identified a marked and consistent upregulation of the serine/threonine kinase *CAMK2A* as a potential contributor to injury induced persistent pain [73] or *STOML3* as a potent modulator of mechanical hypersensitivity [74], while different solute carriers involved in neurotransmitter transport were downregulated (e.g., *SLC6A17*). Although most neuronal injury and neuroinflammatory genes plateaued after removal of paclitaxel, the growth factor *FGF16* and the multidrug transporter *ABCB1* further increased in the *late phase* as a potential delayed tissue remodeling [75] or drug efflux response [76] of early recovery. Importantly, many of the identified differentially expressed genes encode proteins that are transcription factors (e.g., *JUN*, *DDIT3*, *ATF3*, *CREB5*, *EGR1*), protein kinases (*WEE1*, *CAMK2A*) or g-protein coupled receptors (e.g., *CYSLTR2*, *C3AR1*, *CX3CR1*) and hence pharmacologically addressable targets.

Proteomic analysis at incipient neurodegeneration, i.e., 48 h after exposure, largely confirmed differentially expressed genes from RNA sequencing, or at least pointed in the same direction. A notable observation not previously evident in the RNA data, however, is the marked *degradation* of proteins that regulate microtubule dynamics and transport of organelles or macromolecular cargo along the axon. Kinesins are motor proteins driving anterograde axonal transport of, e.g., organelles [77], for which we found a pronounced downregulation of their main neuronal representative, KIF5A. In the same order of magnitude, STMN2 and -3, as regulators of microtubule dynamics, exhibited a marked decrease. In this context, important neuronal scaffold proteins organizing lysosome and phagosome transport along the axons were also reduced (e.g., JIP3, RETREG1). The clinical significance of KIF5A dysfunction has been demonstrated in different neurodegenerative [77] and neuro-inflammatory diseases [78], such as hereditary spastic paraplegia (HSP), axonal Charcot-Marie-Tooth disease type 2 (CMT2) [79] or familial amyotrophic lateral sclerosis [80, 81], while STMN2 and -3 mutations are associated with Alzheimer’s disease [82], sensory and motor neuron degeneration [83, 84] or motor neuron disease [83, 85]. The finding of such strongly downregulated proteins of axon dynamics and transport may explain, at least in part, the dying-back process of long projection axons typically observed in CIPN and its clinical correlate of sensory loss in stocking and glove distribution.

Last, we investigated the lipidome of iPSC-DSN as we found key enzymes of lipid metabolism to be downregulated upon paclitaxel exposure, and as deregulated membrane lipid composition may further contribute to decreased axon stability and axon degeneration. General lipid composition in iPSC-DSN showed the highest abundance for phosphatidylcholines and phosphatidylethanolamines, followed by sphingomyelins, ceramides, and triacylglycerols, which is in line with animal-based literature on DRG [86], while data on human DRG or iPSC-DSN are sparse. 48 h after paclitaxel exposure, we found a tendency of the main lipid classes PC and SM to be downregulated, statistically significantly for SM(26:0) and DAG(12:0/18:1), while TAG were statistically significantly upregulated. Altered neuronal lipid composition is important as membrane fluidity, axonal integrity, and nervous system signaling greatly depend on correct lipid composition [87, 88], and failure of axon regeneration upon nerve injury has been attributed to deregulated lipid biosynthesis such as terpenoid backbone synthesis involving genes of the cholesterol biosynthesis [89]. Whereas changes in lipidomic metabolism were remarkable in the transcriptome and proteome datasets, we assume that the only modestly declined PC and SM may have been detected in a still early phase of neurodegeneration, potentially contributing to the axonal degeneration we observe at later timepoints. The fact that TAGs were upregulated in contrast to the other lipid classes makes this finding unlikely to be a mere sign of energy sequestration, but potentially another marker (or even driver) of inflammation [90, 91].

While we found neuroinflammatory signatures in this reductionist iPSC-based sensory neuron system, translational relevance should be further validated in neuronal co-culture systems harboring glia [92] or in primary human neuronal cells from e.g., tissue donors [25, 52, 93]. Despite robust neurotoxic effects being found across all cell lines, future studies with a larger cohort of patient-specific iPSC-DSN should address the question of whether iPSC-DSN may reproduce the susceptibility to neurotoxicity in vitro, as known for the predilection to cardiotoxicity [94], elucidate the underlying mechanisms and biomarkers differentiating patients with severe CIPN from unaffected individuals, and identify which intervention may help prevent it.

## Conclusions

In summary, this study provides comprehensive insights into the molecular pathways underlying toxic neurodegeneration of CIPN, pointing towards deregulated pathways of the neuronal stress response, apoptosis, lipid metabolism, neuro-inflammation, and nociception. Temporal dynamics of these changes imply early and transient deregulation of mitochondrial gene sets, followed by the upregulation of the transcription factor *JUN* as an early immediate gene and potential inducer of the shift in the ratio of pro- and anti-apoptotic genes, favoring neuronal cell death. These alterations are accompanied by signatures of neuro-inflammation and nociception, as well as markedly degraded proteins of axon transport, microtubule dynamics, and scaffold proteins that may, together with disrupted membrane lipid composition, be correlates of the toxic neuro-axonal degeneration of CIPN. Intriguingly, many of these mechanisms seem conserved between different neurodegenerative and neuroinflammatory diseases, suggesting that upstream modulation in this cascade has immense potential for preventing neuroaxonal damage.

## Methods

### iPSC lines

Experiments from this study were conducted in sensory neurons derived from five distinct iPSC lines. Four cell lines (BIHi263-A, BIHi264-A, BIHi272-A, BIHi273-A) originated from female breast cancer patients (ClinicalTrials identifier NCT02753036; ethics approval at Charité [EA4/069/14], referenced in [36]) that had received paclitaxel chemotherapy and underwent detailed neurological phenotyping (Total Neuropathy Score-reduced, TNSr, and CIPN20 questionnaires [36]). Two of these donors (BIHi264-A and BIHi273-A) had clinically manifest CIPN, whereas two (BIHi263-A and BIHi272-A) were unaffected. The fifth line was derived from a male healthy donor (BIHi005-A), providing a heterogeneous phenotypic background (Suppl. Table 1). Written informed consent was obtained from all individuals prior to sample acquisition. iPSCs were reprogrammed from peripheral mononuclear blood cells (PBMC) or dermal fibroblasts (only BIHi005-A) using Sendai viral vectors, as detailed in [95–97]. iPSCs were tested for the absence of the reprogramming vector with immunofluorescence staining for pluripotency markers, in-vitro directed differentiation into the three germ layers, karyotyping using SNP arrays, and g banding and absence of mycoplasma contamination [95, 96]. iPSCs were maintained on growth factor-reduced Geltrex (Gibco) in E8 media, which was exchanged daily. Cells were enzymatically clump-passaged every 2–4 days when >70% confluency was reached using EDTA (UltraPure™ 0.5 M EDTA, Thermo Fisher).

### Sensory neuron differentiation and maintenance

Differentiation was performed as described previously [34] according to the protocol of Chambers et al. [29] with modifications in media formulations according to the s.c. P2 protocol by *Schwartzentruber* et al. [98]. In brief, >70% confluent clump-passaged iPSCs were single-cell seeded at a density between 200,000 and 400,000/well into 6-well plates (Falcon) coated with growth factor reduced Geltrex (Gibco) and cultivated in E8 media until 70–80% confluency was achieved, which usually required 48–72 h. On *day 0*, neural induction was commenced by replacing E8 media with Knockout Serum Replacement Media (500 ml DMEM-KO [Gibco]) supplemented with 130 ml CTS KnockOut SR XenoFree Medium (Gibco), 1x MEM-Non-essential Amino Acid Solution (Sigma), 1× Glutamax (Gibco) and 0.01 mM β-Mercapto-ethanol (Gibco) containing the two small molecule inhibitors ‘2i’: 100 nM LDN-193189 (Sigma) and 10 µM SB-431542 (Peprotech) to drive neuroectoderm differentiation. On *day 2*, ‘3i’ consisting of 3 µM CHIR99021 (Sigma), 10 µM DAPT (Sigma), and 10 µM SU5402 (Sigma) was added to promote neural crest specification. *On day 4*, N2B27 media (500 ml Neurobasal-Medium [Gibco], 5 ml of N-2 (100×) supplement [Gibco], 10 ml of B27-supplement (50×) without Vitamin A [Gibco], 1× Glutamax, and 0.01 mM β-Mercapto-ethanol [Sigma]) was progressively phased in. From *day 6* onwards, ‘2i’ addition was ceased but ‘3i’ continued to be added until Day 11. On *day 11*, iPSC-DSN were reseeded using TrypLe select (Gibco) for dissociation and resuspended in N2B27 maintenance media containing 10 μM ROCK inhibitor, at densities of 48,000/well into geltrex-coated 96-well plates for cell viability/cytotoxicity assays and MTT live assays, 1mio/well into 6-well plates for transcriptomic, proteomic and lipidomic experiments, 30,000 cells/well in 48-well MEA plates for electrophysiological assessment, 100,000/well in 8-chamber-slides (ibidi) for calcium imaging or staining and in a droplet at 50,000–100,000cells/well onto coverslips for patch-clamp experiments. ROCK inhibitor containing N2B27 media was removed 24 h after reseeding and replaced with N2B27 supplemented with BDNF, GDNF, βNGF and NT3 (all at 25 ng/ml, Peprotech). On day 14, cells were treated with 1 µg/ml Mitomycin C (Sigma) for 2 h to eliminate a few dividing non-neuronal cells. From day 14 onwards, 1× Pen/Strep (Gibco) was also supplemented. iPSC-DSN were maturated for at least 4 more weeks for viability assays (>d40), at least 40 more days for stainings, calcium imaging and patch-clamp experiments (>d50) and 50 more days for multi-electrode-arrays, transcriptome and proteome analyses (>d60) for relative functional maturity [30]. Half media change was performed every 3–4 days with N2B27 supplemented with BDNF, GDNF, βNGF, and NT3 (all at 25 ng/ml, Peprotech; summary of differentiation timeline in Suppl. Fig. 8).

### Immunohistochemical stainings

iPSC-DSN >d50 were stained on geltrex coated coverslips or 8-chamber-slides. Cells were fixed with 2% paraformaldehyde (prepared from ROTI®Histofix) for 15 min, rinsed twice and incubated for 1 h at room temperature with blocking buffer consisting of 1× PBS with Ca^2+^ and Mg^2+^ (PBS+/+), 1% bovine serum albumin (Sigma), 10% normal goat serum (abcam) and 0.1% Triton X (Sigma). The following primary antibodies were diluted in 1% BSA: 1:1000 Peripherin (rabbit, ThermoFisher) and 1:1000 NFL (mouse, Uman Diagnostics), 1:100 TRPM8 (rabbit, Abcam), 1:100 TRPV4 (rabbit, LifeSpan). After incubation for 24 h at 4 °C, cells were washed 3× with 1× PBS +/+ and incubated for 1 h at room temperature with 1:600 secondary antibodies diluted in 1% BSA, using goat anti-rabbit Alexa 488 (Invitrogen) or goat anti-mouse Alexa 488 (Invitrogen) if single stained, or in combination with goat anti-mouse Alexa 568 (Invitrogen) if double-stained, and washed 3× with 1× PBS +/+. Nuclei were stained with 10 μM DRAQ5 (Thermo Scientific) by 30 min incubation, washed once with 1× PBS +/+ and mounted with ProLong™ Gold Antifade Mountant DAPI (Molecular Probes). Stainings were visualized on a Leica TCS SP II with a Leica DFC3000G camera fitted with 10×, 20×, 40×, and 63× objectives.

### Compound preparation

Paclitaxel (Adipogen) was dissolved in the appropriate amount of DMSO to reach a 6 mM stock solution, which was further diluted in N2B27 media to reach the final concentrations 100pM, 1 nM, 10 nM, 100 nM, 1 µM and 10 µM. Concentrations of the vehicles were adapted to the amount of DMSO in which the highest paclitaxel dosage was dissolved, e.g., 1/600 DMSO for cell viability experiments (as paclitaxel concentrations reached a maximum of 10 µM for viability curve calculation), or 1/60,000 for RNA sequencing, proteomic, and lipidomic experiments (paclitaxel concentration of 100 nM). All solutions were prepared on the day of experimentation.

### Cell viability and cytotoxicity assays

For the calculation of cell viability, RealTime-Glo™ MT Cell Viability Assays (Promega) were performed in white wall, clear bottom 96-well imaging plates (Greiner), according to the manufacturer’s instructions. Luminescence intensities were measured every 12 h up to 108 h using the Berthold TriStar LB 941 luminescence reader, with Mikrowin Software, and all values were background subtracted and normalized to the individual well baseline and vehicle control [99].

### Transcriptome sequencing analyses

#### Experimental setup

Seventy-two samples were included in RNA sequencing analyses from 12 different experimental units (plates): Seven time points from BIHi264-A (7 × 6-well plates) and 48-h samples from five cell lines (5 × 6-well plates). iPSC-DSN were maintained in geltrex coated 6-well plates at 10^6^ cells/well as described above. After maturation for >60 d, three wells were either treated with DMSO at 1/60,000 or paclitaxel at 100 nM, respectively. Investigated time points were 48 h of paclitaxel incubation for all five cell lines (30 samples), and for BIHi264-A, the additional time points of 2 h, 6 h, 12 h, 24 h and 24 h or 120 h following removal of the drug after 48 h incubation (s.c. 72 h and 168 h timepoints; in total, 42 samples). RNA was harvested with the Aurum™ Total RNA Mini Kit according to manufacturers’ instructions. RNA sequencing was performed by Brooks Life Sciences Genewiz® with PolyA selection for RNA removal, 2 × 150 bp sequencing configuration and 20–30 million reads per sample. *RNA Library Preparation and NovaSeq Sequencing by AzentaLifeSciences Genewiz:* RNA samples were quantified using Qubit 4.0 Fluorometer (Life Technologies) and RNA integrity was checked with an RNA Kit on Agilent 5300 Fragment Analyzer (Agilent Technologies). RNA sequencing libraries were prepared using the NEBNext Ultra RNA Library Prep Kit for Illumina following the manufacturer’s instructions (NEB, Ipswich). Briefly, mRNAs were first enriched with Oligo(dT) beads. Enriched mRNAs were fragmented for 15 min at 94 °C. First-strand and second strand cDNAs were subsequently synthesized. cDNA fragments were end repaired and adenylated at 3’ends, and universal adapters were ligated to cDNA fragments, followed by index addition and library enrichment by limited-cycle PCR. Sequencing libraries were validated using NGS Kit on the Agilent 5300 Fragment Analyzer (Agilent Technologies), and quantified by using Qubit 4.0 Fluorometer (Invitrogen). The sequencing libraries were multiplexed and loaded on the flowcell on the Illumina NovaSeq 6000 instrument according to manufacturer’s instructions. The samples were sequenced using a 2 × 150 Pair-End (PE) configuration v1.5. Image analysis and basecalling were conducted by the NovaSeq Control Software v1.7 on the NovaSeq instrument. Raw sequence data (.bcl files) generated from Illumina NovaSeq was converted into fastq files and de-multiplexed using Illumina bcl2fastq program version 2.20. One mismatch was tolerated for index sequence identification.

#### Computational methods

RNA Seq reads were mapped to the human genome (GRCh38.p7) with STAR-version 2.7.3a [100] using the default parameters. Reads were assigned to genes with featureCounts version 2.0.0 [101] with the following parameters: -t exon -g gene_id -s 0 -p, gene annotation—Gencode GRCh38/v25. The differential expression analysis was carried out with DESeq2-version 1.32.0 [102] using the default parameters. We kept genes that have at least five reads in at least three samples. Gene set enrichment analysis was carried out with R/tmod package version 0.50.07 using MSigDB gene sets [103].

### Proteomic analyses

Confirmatory proteomic analyses were performed in six samples of iPSC-DSN BIHi264-A d68 (3 treated with DMSO at 1/60,000 and 3 with paclitaxel at 100 nM for 48 h) after a microscopic quality check of each well and exclusion of proliferating cells. For cell pellet collection, supernatant was aspirated and cell lawn washed with cold PBS +/+, aspirated and discarded. Then, 500 µl of cold PBS +/+ were added onto the cell lawn and cell pellet aspirated with 1 ml pipette tip, supported by pipetting up and down and scrapping. The suspension was then transferred into an aliquot and centrifuged for 5 min at 1000×*g* at 4 °C. The supernatant was aspirated from the aliquots and cell pellets snap frozen in liquid nitrogen and then stored at −80° until analysis. For proteomic profiling by mass spectrometry, cell pellets were resuspended in lysis buffer (1% sodium deoxycholate, 150 mM NaCl, 10 mM dithiothreitol (DTT), 40 mM Chloroacetamide (CAA), 100 mM Tris-HCl pH 8), heated for 10 min at 95 °C and treated with Benzonase® for 30 min at 37 °C. 50 µg protein per sample was digested with endopeptidase LysC (Wako) and sequence-grade trypsin (Promega) at 37 °C with an enzyme-to-protein ratio of 1:25. The resulting peptides were cleaned-up and desalted on C18 SepPak columns (Waters, 100 mg/1cc), resolved in 50 mM HEPES (pH 8) and labeled with 16-plex tandem mass tag (Fisher Scientific) reagents following the vendor’s instructions. All peptide samples were combined, desalted and fractionated by high-pH reversed phase off-line chromatography (1290 Infinity, Agilent), followed by pooling into 30 fractions. Each fraction was analyzed separately by LC-MS/MS. Peptides, reconstituted in 3% acetonitrile with 0.1% formic acid, were separated on a reversed-phase column (20 cm, 75 µm inner diameter, ReproSil-Pur C18-AQ 1.9 µm resin [Dr. Maisch GmbH]), using a 98 min gradient with a 250 nl/min flow on a High Performance Liquid Chromatography (HPLC) system (Thermo Fisher Scientific) and directly measured on an Q Exactive HF-X instrument (Thermo Fisher Scientific). The mass spectrometer was operated in data-dependent acquisition mode using the following settings: full-scan automatic gain control (AGC) target 3 × 10^6^ at 60 K resolution; scan range 350–1500 *m*/*z*; Orbitrap full-scan maximum injection time 10 ms; MS/MS scan AGC target of 1 × 10^5^ at 45 K resolution; maximum injection time 86 ms; normalized collision energy of 30 and dynamic exclusion time of 30 s; precursor charge state 2–6; 20 MS2 scans per full scan. RAW data were analyzed with MaxQuant software package (v 1.6.10.43) using the Uniprot databases for human (UP000005640_2019_07). The search included variable modifications of methionine oxidation and N-terminal acetylation, deamidation (N and Q) and fixed modification of carbamidomethyl cysteine. Reporter ion MS2 for TMT16 was selected (internal and N-terminal) and TMT batch specific corrections factors were specified. The FDR was set to 1% for peptide and protein identifications. Unique and razor peptides were included for quantification. The resulting text files were filtered to exclude reverse database hits, potential contaminants, proteins identified by site only and with less than two MS/MS counts. For statistical data analysis, the log2-transformed and normalized reporter ion intensities were used. Differences in protein abundance between experimental groups were calculated using Student’s *t*-test. Signals passing the significance cut-off of FDR 5 or 10 were considered differentially expressed. Gene set enrichment analyses of proteomic data was carried out with cerno test from R/tmod package version 0.50.07 using R/msigdbr gene sets [104].

### Lipidomic analyses

Lipidomics was conducted by BIH Metabolomics. Lipidomic analyses were performed in six samples of iPSC-DSN BIHi264-A > d60 (6 treated with DMSO at 1/60,000 and 6 with paclitaxel at 100 nM for 48 h, pooling of 2 wells resulted in 3 vs 3 samples). iPSC-DSN were first washed by removing media in each well and adding 1 ml wash buffer (140 mM NaCl, 5 mM HEPES, pH 7.4). Next, the wash buffer was removed and replaced with 1 ml ice-cold extraction solvent that consisted of 50% methanol on LC-MS grade water with 2 μg/ml cinnamic acid (intended but not used as an internal standard for polar metabolites). The lysate was transferred to a prechilled Falcon tube. The falcon tube was immediately placed on dry ice. There were two types of quality control samples prepared. Reference quality control (QC) plasma samples were prepared using 100 µl plasma extracted with 4 ml of 50% methanol in water (*v*/*v*) containing cinnamic acid as an internal standard at 2 µg/ml. The subsequent lipid extraction was carried out in glass vials with the addition of chloroform to achieve a ratio of 1:1:1 of methanol:water:chloroform (*v*/*v*/*v*) according to a modified Bligh and Dyer method [105]. Pooled Quality control (QC pool) samples were created by pooling all biological sample extracts. Samples and QC samples were supplemented with the internal standard mixture of the lipidizer platform kit (Sciex), vortexed, and chilled on ice for phase separation. After centrifugation, the polar and lipid fractions were transferred into separate glass vials and lyophilized. Lipids were dissolved in the mobile phase methanol/dichlormethane in the ratio 1:1 (*v*/*v*) with 10 mM ammonium acetate. Direct infusion analysis (FIA) and a multiple reaction monitoring mode (MRM) were carried out with a QTRAP Sciex 5500 mass spectrometer (MS) (Sciex, Framingham, MA, USA). An isocratic flow rate of 35 µl/min was used to equilibrate the autosampler of the Shimadzu Nexera X2 liquid chromatograph (LC) (Shimadzu, Kyoto, Japan). Lipids were analyzed by direct injection using the standard method detailed by Sciex Lipidyzer, including using differential ion mobility spectrometry (DIMS) to separate lipid species [106–110]. The resulting concentrations of lipid species in nmol/ml were processed for data analysis in R Studio using in-house scripts. Pooled sample quality controls (QCs) and plasma QCs injected at the beginning, end, and along the sequence run were used for quality assurance and data filtering. A relative standard deviation of 15% in pooled QCs was accepted as a cut-off for reliably detected lipid species. Samples or species with missing values of more than 50% were filtered out. Filtering and visualization in box plots, heatmaps, correlation plots, principal component analysis (PCA) plots, and circular plots were performed in R Studio and GraphPad Prism.

### Statistical analyses

All data were analyzed using RStudio and GraphPad Prism v8. Data from viability/cytotoxicity assays and multielectrode arrays were checked for Gaussian distribution using the Shapiro–Wilk normality test and histograms. Normally distributed data were analyzed with two-sided *t*-tests, and data not fulfilling the criterion of normal distribution with the Mann–Whitney *U*-test (2 groups). For multiple comparisons, one-way ANOVA was applied and corrected using Holm–Sidak’s multiple comparison test (normally distributed data) or the Kruskal–Wallis test corrected using Dunn’s multiple comparison test (data that were not normally distributed). Dose-response curves were calculated with non-linear regression models using GraphPad Prism (Log inhibitor vs response, three parameters). All experiments were replicated at least three times with at least three technical replicates for each condition (detailed description of sample sizes given in the respective figure legends). Normally distributed data are presented as mean ± SD or 95% confidence interval as stated in the respective figure legends. Statistical analyses of transcriptome, proteome, and lipidome data are given in the respective paragraphs above.

## Supplementary information

Suppl. Figure Captions and Suppl. Methods

Supplementary Figure 1.

Supplementary Figure 2.

Supplementary Figure 3.

Supplementary Figure 4.

Supplementary Figure 5.

Supplementary Figure 6.

Supplementary Figure 7.

Supplementary Figure 8.

Suppl. Table 1.

Suppl. Table 2.

Suppl. Table 3.

Suppl. Table 4.

Suppl. Table 5.

Dataset 1.

## Data Availability

Open data publishing guidelines were followed. All raw data, R scripts for analyses, and GraphPad Prism files are available with this manuscript (folder: CDD_OpenData.zip). RNA sequencing data discussed in this publication have been deposited in NCBI’s Gene Expression Omnibus (GEO) [111] and are accessible under the GEO Series accession number GSE312881. The mass spectrometry proteomics data have been deposited to the ProteomeXchange Consortium via the PRIDE partner repository [112] with the dataset identifier PXD070094. Method descriptions of whole cell patch-clamp, calcium live-cell imaging, and multi-electrode arrays are given in Supplementary Methods. Suppl. Table 5 contains a comprehensive list of all assays, reagents, antibodies, and related materials used.
