# Supplementary material for: Time‑resolved multi-omic analysis of paclitaxel exposure in human iPSC‑derived sensory neurons unveils mechanisms of chemotherapy‑induced peripheral neuropathy
Source: Cell Death Dis. 2026 Feb 10;17(1):211. doi: 10.1038/s41419-026-08445-2 (PMC12921266; doi:10.1038/s41419-026-08445-2)
Supplement: Supplementary file 1 — Suppl. Figure Captions and Suppl. Methods [file 41419_2026_8445_MOESM1_ESM.docx]

**Supplementary Figures – Figure legends and captions**

**Supplementary Figure 1.** *Morphological and functional characteristics of iPSC-DSN.* Few days after replating, early neuronal clusters appeared that formed large ganglia and axonal network in the maturation phase **(A)**. iPSC-DSN expressed the capsaicin receptor transient receptor potential cation channel subfamily V member 4 (TRPV4) and the cold receptor transient receptor potential cation channel 8 (TRPM8, **B**). **(C)** shows whole cell patch clamp recording with the Alexa488 containing intracellular solution. Left: reconstruction of the soma and surrounding neurites. Right: membrane potential changes upon a 1s hyper- or depolarizing current steps, with the red trace representing the response at the action potential threshold current. Note the presence of the prominent sag (Ih current) upon hyperpolarization that is typical for sensory neurons. To assess iPSC-DSN receptor functionality, calcium imaging experiments were conducted in iPSC-DSN >d50. We observed 72 ± 39% (SD) positive responses to 100µM adenosine trisphosphate (ATP), an agonist of P2X/P2Y purinoreceptors, 19 ± 17% responses to 10µM capsaicin, an agonist of the TRPV1 receptor, and 40 ± 27% positive responses on stimulation with 10µM icilin, a TRPM8 receptor, with typical response curves, respectively **(D-E)**. To assess electrophysiological activity, iPSC-DSN were densely grown on MEA plates until maturation d65 **(F)**, exhibiting spikes (>6 SD voltage change from baseline) and bursts **(G)**. In the observation period of 72h, mean firing rate and burst frequency significantly peaked four hours after media change and normalized to baseline 24h thereafter (mean firing rate: 2.8 ± 1.2Hz [baseline] vs. 7.3 ± 3.8Hz [4h], *p = 0.017*, Kruskal-Wallis test with Dunn’s correction; burst frequency: 0.06 ± 0.02 Hz [baseline] vs. 0.18 ± 0.07 Hz [4h], *p < 0.001*, ANOVA), while the number of spikes/burst as a measure of active iPSC-DSN in a cluster remained unaffected by the media change (MC, # spikes/burst: 27 ± 13, **H**). A-H show representative experiments from iPSC-DSN BIHi264-A. All applied cell lines of this study were characterized and showed comparable sensory neuron functionality.

**Supplementary Figure 2.** *Viability assay,* *gene correlation across cell lines and network analyses.* **(A)** MTT-live cell assays confirmed a dose- and time-dependent decline of cell viabilities initiating at 48h, with an IC50 of about 100nM which corresponds to the clinically applied steady state concentration. Plateau was reached for 100nM at 84h (mean with 95% confidence interval), while supratherapeutic dosages led to a further decline without plateauing. **(B)** Concordance-discordance plots (disco) showed a very high correlation between the deregulated genes across, e.g., the cell lines, with highest consistency for, e.g., *HRK*, *MMP10*, *CALCB*, *IL31RA, CYSLTR2, JUN, ATF3, CASP3, ABCB1* and *HMGCS1*. **(C)** Amongst the 50 most-robustly deregulated genes across the cell lines BIHi005-A and BIHi264-A, string-db network analyses predicted central mechanistic roles for *JUN*, *CASP3* and *HMGCR*. A: Experiments pooled from 3 experimental units (plates) with 3 technical replicates per plate and condition, all from BIHi264-A. B-C: 6 replicates (wells) of iPSC-DSN for each cell line were included, 3 treated with 100nM paclitaxel and 3 with DMSO, respectively.

**Supplementary Figure 3.** *Gene* *co-expression network of consistently deregulated genes across cell lines.* **(A)** Connectivity heatmap of pairwise Pearson-correlation coefficients |r| > 0.80 of differentially expressed genes upon paclitaxel treatment (log2FoldChange >0.5, statistically significant differential expression in pooled data set and in at least one iPSC-DSN cell line). The heatmap shows the top 60 genes with the highest overall connectivity, indicating genes that exhibit highly similar differential expression patterns across samples. **(B)** Pearson co-expression network of differentially expressed genes upon paclitaxel treatment. Nodes are sized by the number of iPSC-DSN cell lines in which the gene was significantly differentially expressed (Overlaps: 1–5) and colored by log₂fold change (red: up, blue: down). Lines connect gene pairs with Pearson |r| > 0.80 (red = positive correlation, blue negative). Layout uses a force-directed algorithm, i.e., genes that cluster together have similar differential expression profiles. Dashed circle indicates early significant differential gene expression from 12h onwards (*JUN*). Data reflect pooled transcriptomic profiles from 30 wells of 5 genetically distinct iPSC-derived sensory neuron (iPSC‑DSN) lines (with 3 paclitaxel vs. 3 DMSO for each cell line).

**Supplementary Figure 4.** *Temporal transcriptomic profiling during paclitaxel induced neurotoxic degeneration - gene sets of interest in the course of time.* **(A)** Panel and evidence plots outlined an early but transient deregulation of mitochondrial gene sets as early as 6h after paclitaxel incubation **(B)**, followed by deregulated transport and microtubule associated **(C)** and markedly downregulated cholesterol biosynthesis gene sets at 24h **(D)** as well as gene sets of the cellular stress response at 48h **(E)**. Although a reductionist pure neuronal culture system, remarkably, we found gene set signatures of neuroinflammation, e.g., of glial cell migration in the late phase of injury **(F)**. 5d after removal of paclitaxel, we observed gene sets indicative of disturbed neurotransmitter loading into synaptic vesicles as another hint of disturbed neuronal transmission **(G)**. All panel plots of the different gene ontology platforms are given in CDD_OpenData.zip. A-G: 42 replicates (wells) of iPSC-DSN were used (all BIHi264-A) from 7 experimental units (plates, 1 for each timepoint, with 3 paclitaxel vs. 3 DMSO-treated wells, respectively).

**Supplementary Figure 5.** *Transcriptomic signatures relevant to the pathophysiology of chemotherapy-induced peripheral neuropathy (CIPN).* After 2-6h of paclitaxel exposure, no significantly deregulated genes were found **(A-B)**. **(C)** shows the differential expression of genes five days after removal of the drug. The differential expression of lipid biosynthesis genes, the multidrug transporter *ABCB1* and the growth factor *FGF16* further increased in magnitude in the *early recovery phase* after 48h exposure to paclitaxel.

**Supplementary Figure 6.** *Transcriptomic signatures relevant to the pathophysiology of chemotherapy-induced peripheral neuropathy (CIPN).* **(A)** The number of differentially expressed genes gradually increased during paclitaxel exposure even after removal of the drug (red: upregulated genes, blue: downregulated genes). The most consistently deregulated genes were assigned to mechanistic groups relevant in CIPN pathogenesis **(B-F)**. **(B)** Out of the cellular stress response associated genes, *JUN* steadily increased from 12h onwards, plateauing earlier at 24h. **(C)** Apoptosis and neuroaxonal injury associated signatures as well as neuroinflammatory genes **(D)** plateaued between 48 and 72h which coincides with the observed decline of cell viabilities. As an exception in these groups of mechanisms, lipid metabolism associated genes **(E)**, *FGF16* and *ABCB1* **(F)** steadily increased in absolute log2FC even 5d after removal of the drug (168h). A-F: 42 replicates (wells) of iPSC-DSN were used (all BIHi264-A) from 7 experimental units (plates, 1 for each timepoint, 3 paclitaxel vs. 3 DMSO-treated wells, respectively). 1 replicate had to be excluded prior to analyses due to detachment of cell lawn and low RNA quality (168h timepoint).

**Supplementary Figure 7.** *Principal component analyses of lipidomic results.* 12 replicates (wells) of iPSC-DSN were included (all BIHi264-A), with 6 paclitaxel vs. 6 DMSO-treated wells (2 wells pooled for 1 analysis).

**Supplementary Figure 8.** *Illustration of* *iPSC-DSN differentiation.* **(A-B)** Differentiation protocol adapted from [1] after [2]. **(C)** and **(D)** show media formulations according to the s.c. P2-protocol from [3].

## **Supplementary Methods**

## Whole cell patch-clamp

Whole cell patch-clamp experiments were performed between maturation day 55 and 65. Recordings were performed in a submerged recording chamber mounted on an Olympus BX-51WI microscope perfused with a carbogen saturated (95 % CO_2_, 5 % O_2_) extracellular solution at room temperature containing in mM: NaCl 129, KCl 3, NaH_2_PO_4_ 1.25, MgSO_4_ 1.8, CaCl_2_ 1.6, NaHCO_3_ 26, and glucose 10, pH 7.3. Borosilicate glass pipettes (5-8 MOhm) were filled with intracellular solution containing in mM: K-gluconate, 135, HEPES 10, KCL 6, MgCl_2_ 2, EGTA 0.2, Na_2_ATP 2, Na_2_GTP 0.5, Na_2_-Phosphocreatine 5, Alexa488 0.1, 280-290 mOsm, pH 7.3. Cells were visualized by a Yokogawa spinning disk confocal microscope equipped with a digital camera (Andor iXonDU867). Recordings were performed from n=19 cells using a Multiclamp 700B amplifier (Molecular Devices). Low-pass filtering was applied at 3 kHz using the amplifiers built in Bessel filter and digitized with a Digidata 1440 (Molecular Devices) at a sampling rate of 10 kHz. The pClamp 10.4 software package (Molecular Devices) was applied for data acquisition and analysis [4]. *Intrinsic electrophysiological properties:* Zero net current membrane potential (Em), membrane resistance (Rm) and access resistance (Ra), were determined automatically in voltage clamp immediately after breakthrough. The cells had a resting Em of -60 ± 6.1 mV, Rm 162.7 ± 90.1 MOhm, Ra: 14.3 ± 6 mOhm. In current clamp mode, bridge balance was automatically compensated before recording with 500 ms long hyperpolarizing and depolarizing steps. Instantaneous frequency (45.9 ± 17.3 Hz) was obtained at the current step with highest frequency without depolarization block. The hyperpolarization-activated, cyclic nucleotide-gated current (I*_h_*), is typically present on DRG neurons and contributes to the control of their excitability. The sag ratio (0.9 ± 0.04) was defined as the ratio between the steady state decrease in the voltage and the largest decrease in voltage following a -150 pA hyperpolarizing current step. AP full-width-at-half-maximum (1.9 ± 0.6 ms) and amplitude (69.3 ± 15.8 mV) were from Aps induced by slow current injection (rheobase current), Suppl. Figure 1C*.*

## *Calcium live-cell imaging*

Calcium imaging was performed as described previously [5]. iPSC-DSN were loaded with Fluo-4 AM (Molecular Probes) dissolved in 0.02% Pluronic F-127 (Life Technologies) for 30 min at 37°C in a standard HEPES buffered solution prepared as 130 mM NaCl, 4.7 mM KCl, 1 mM MgSO4, 1.2 mMKH2PO4, 1.3 mM CaCl_2_, 20 mM HEPES and 5mM glucose, pH 7.4. Cell cultures were then washed with standard HEPES buffered solution. Then, 8-Chamberslides (ibidi) were placed in Leica TCS SP II with a Leica DFC3000G camera fitted with 10x, 20x, 40x and 63x objectives, of which commonly 10x - 20x was used. Fluorescence signals were detected at 1 Hz, and data processed using Leica LASX imaging software. Experiments were performed at room temperature. Fluo-4 intensities were calculated for all regions of interest (ROI) after background subtraction. Stock solutions were diluted in HEPES buffered solution and manually added in a 1:10 ratio to the wells to reach a final concentration of 100 μM ATP (Tocris), 10 μM icilin (Tocris) and 10 μM capsaicin (Hello Bio), and positive responses (defined as an increase of >20 % of baseline) were observed for all drugs, see Suppl. Fig. 1D.

## Multielectrode array experiments

Experiments were conducted with iPSC-DSN >d60 in 48-well multielectrode-array plates with 16 electrodes/well in Maestro Pro AxionBiosystems multi electro array system. *Coating:* MEA plates were coated with polyethylenimin (PEI, Sigma) 0.07% in borate buffer (ThermoScientific), applying a droplet of 7µl directly to the center of each well, and incubated for 1h at 37°C. Afterwards, wells were carefully rinsed 4x with 400µl/well aqua dest without disturbing the coating, and dried overnight (e.g, under the bench) according to the manufacturer’s instructions. Wells were then geltrex-coated with a 7-8µl droplet in the center of each well, avoiding the droplet to spill over the reference electrodes. Geltrex was left to dry for one hour under the bench. *iPSC-DSN seeding:* d11 iPSC-DSN were reseeded in a 7µl droplet of 30.000cells into the center of each well and left to settle down for about 30-45min at 37° and 5% CO_2_ until 400µl of N2B27 media with growth factors were added. Cells were maintained and matured as described above. *Experimental setup:* On maturation day 63, experiments were conducted at 37°, 5% CO_2._ Baseline spontaneous neural activity was measured before media change (baseline before MC). Then, paclitaxel at 1nM, 10nM and 100nM vs. DMSO (1/60.000) were washed in, with 6 technical replicates (wells) for each condition (for Suppl. Fig. 1F-H, all experiments were conducted in BIHi264-A). Neural activity was measured for 10min every 4h for the first 24h and then every 12h until 72h as well as 5d after washout of the drug (s.c. 192h timepoint). The 00h timepoint was measured 30min after the drugs had been washed-in to ensure regaining of 37°C. The software Axion Biosystems AxIS Navigator has been used for analyses. Heatmaps and raster plots are derived from representative screenshots from the software at given time points. *AxIS Navigator adjustments:* The default mode for measuring spontaneous neural activity was used. *MEA configurations:* Neural Real-Time, Spontaneous; spike detection at > 6SDs from baseline, Electrode Burst Settings: Interspike-Interval (ISI) threshold, Min # of Spikes 5, Max ISI (ms) 100; Network Burst Settings: Algorithm ISI Threshold Min # of Spikes 50, Max ISI (ms) 100, Min # of Electrodes (%) 35, Synchrony Settings Synchrony Window 20; Average Network Burst Analysis Settings: Number of network bursts vary by well, Network Burst Window Start (ms) 0, Network Burst Window End (ms) 500, Bin Size (ms) 1.

## Sample size justification

Sample sizes were estimated based on our prior works (Schinke et al., 2021) and agreed on with the Core Unit Bioinformatics and the Core Unit pluripotent Stem Cells and Organoids at the Berlin Institute of Health.

**References**

1. Schinke C, Fernandez Vallone V, Ivanov A, Peng Y, Körtvelyessy P, Nolte L, Huehnchen P, Beule D, Stachelscheid H, Boehmerle W *et al*: **Dataset for: Modeling chemotherapy induced neurotoxicity with human induced pluripotent stem cell (iPSC)-derived sensory neurons**. *Data Brief* 2021, **38**:107320.

2. Chambers SM, Qi Y, Mica Y, Lee G, Zhang X-J, Niu L, Bilsland J, Cao L, Stevens E, Whiting P *et al*: **Combined small-molecule inhibition accelerates developmental timing and converts human pluripotent stem cells into nociceptors**. *Nature biotechnology* 2012, **30**(7):715-720.

3. Schwartzentruber J, Foskolou S, Kilpinen H, Rodrigues J, Alasoo K, Knights AJ, Patel M, Goncalves A, Ferreira R, Benn CL *et al*: **Molecular and functional variation in iPSC-derived sensory neurons**. *Nature genetics* 2018, **50**(1):54-61.

4. Peng Y, Barreda Tomas FJ, Klisch C, Vida I, Geiger JRP: **Layer-Specific Organization of Local Excitatory and Inhibitory Synaptic Connectivity in the Rat Presubiculum**. *Cereb Cortex* 2017, **27**(4):2435-2452.

5. von der Ahe D, Huehnchen P, Balkaya M, Peruzzaro S, Endres M, Boehmerle W: **Suramin-Induced Neurotoxicity: Preclinical Models and Neuroprotective Strategies**. *Molecules (Basel, Switzerland)* 2018, **23**(2).
