# Supplementary figures and images for: Time‑resolved multi-omic analysis of paclitaxel exposure in human iPSC‑derived sensory neurons unveils mechanisms of chemotherapy‑induced peripheral neuropathy
Source: Cell Death Dis. 2026 Feb 10;17(1):211. doi: 10.1038/s41419-026-08445-2 (PMC12921266; doi:10.1038/s41419-026-08445-2)

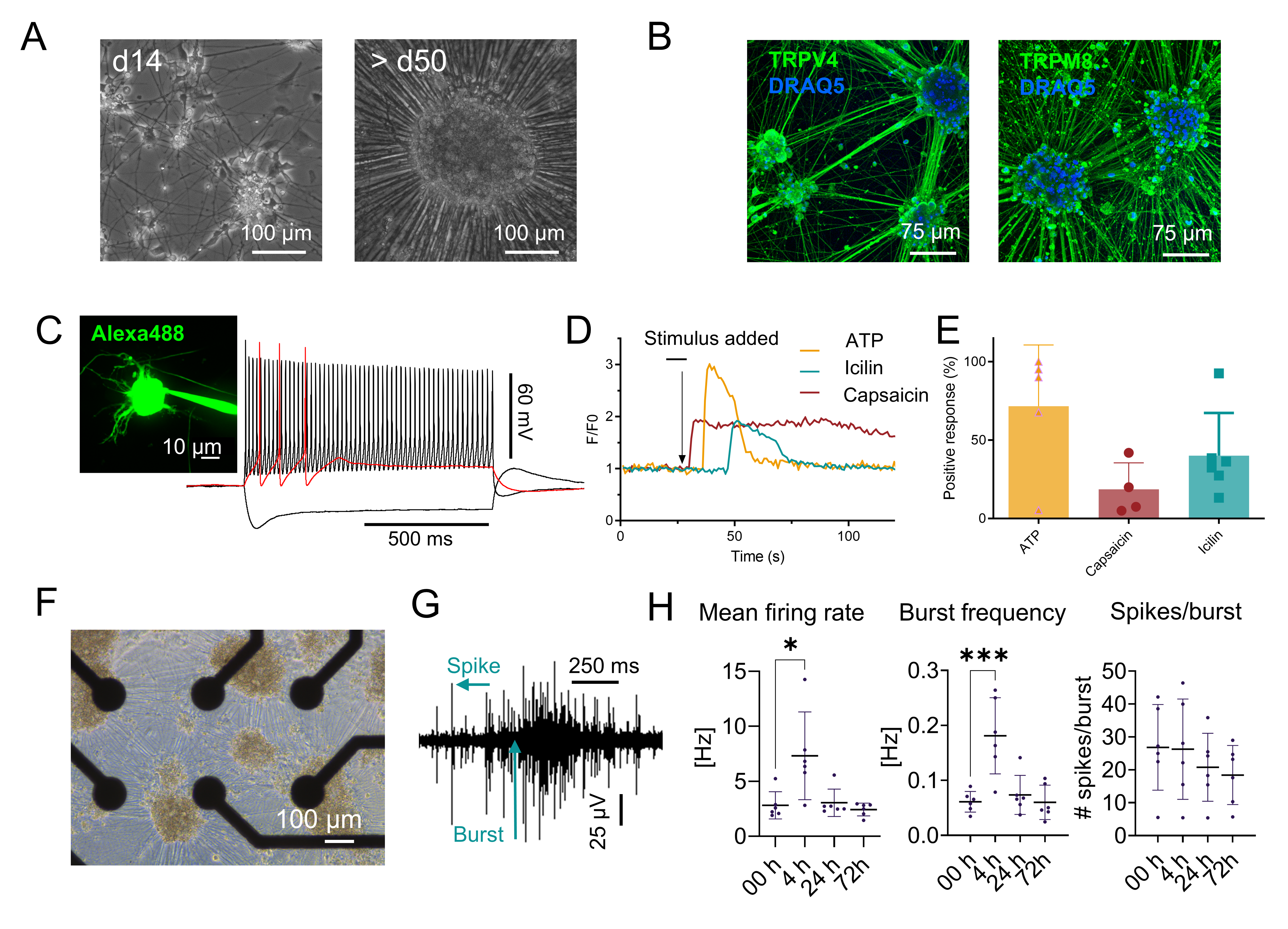

Supplement: Supplementary file 2 — Supplementary Figure 1. [file 41419_2026_8445_MOESM2_ESM.tif]

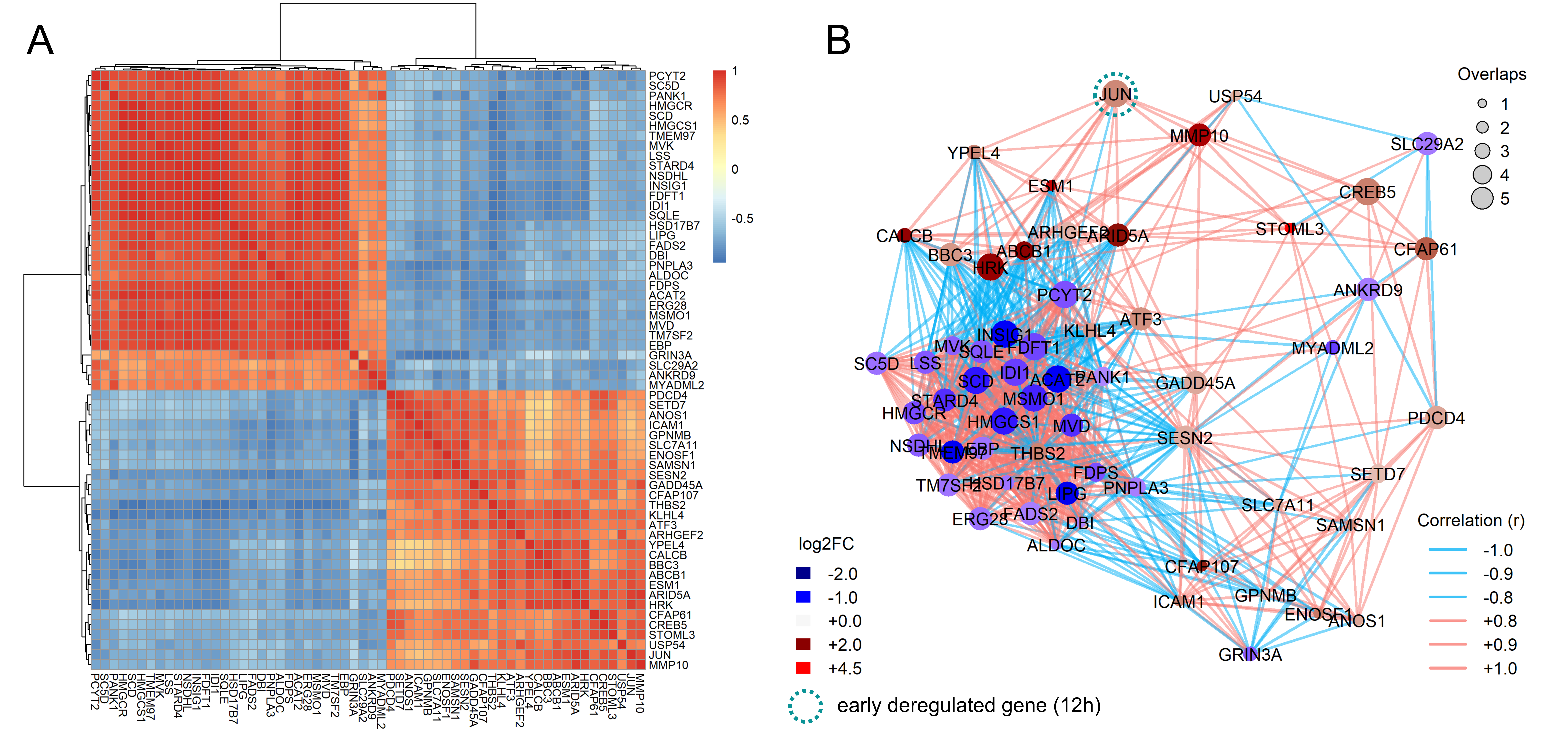

Supplement: Supplementary file 4 — Supplementary Figure 3. [file 41419_2026_8445_MOESM4_ESM.tif]

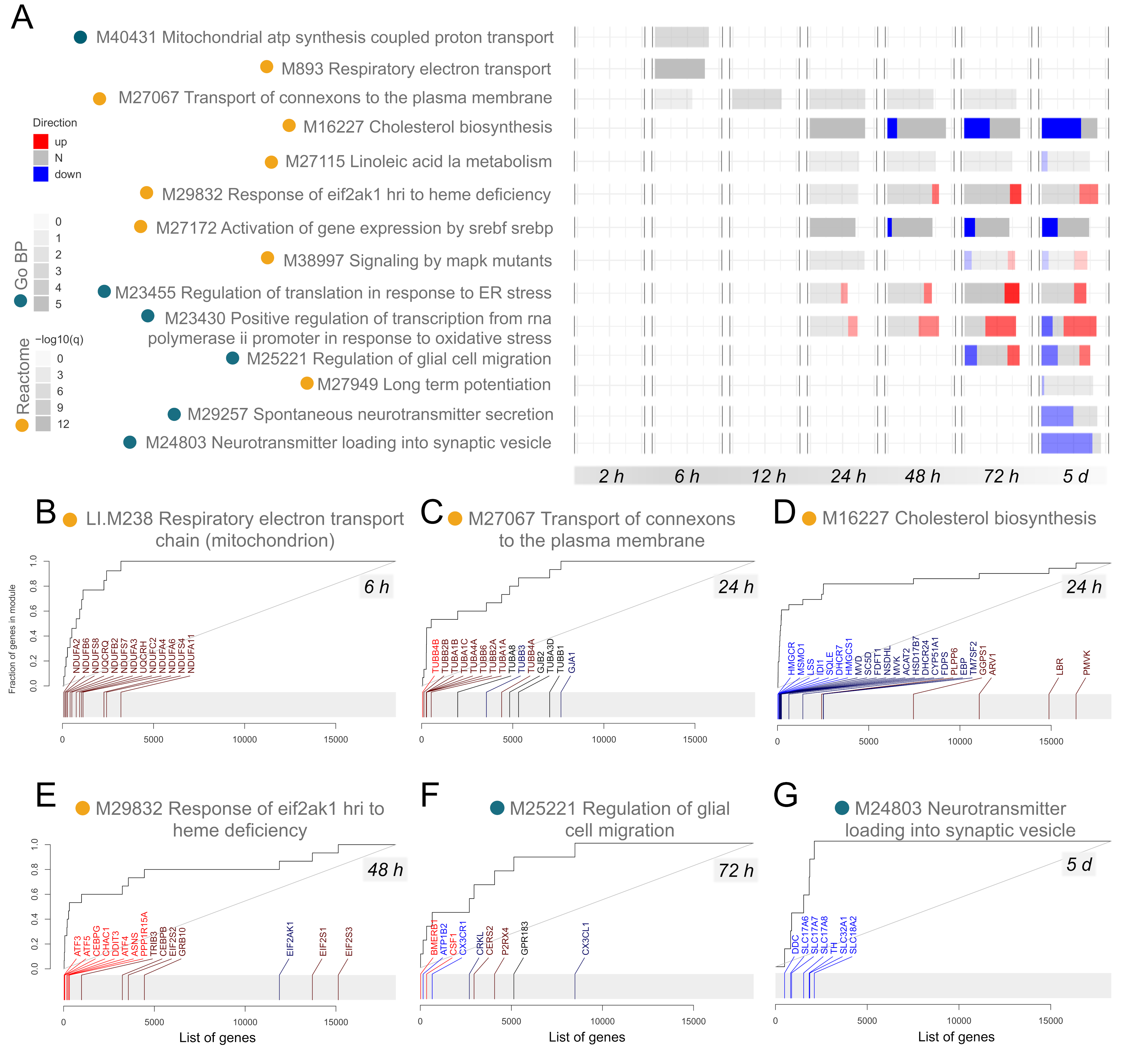

Supplement: Supplementary file 5 — Supplementary Figure 4. [file 41419_2026_8445_MOESM5_ESM.jpg]

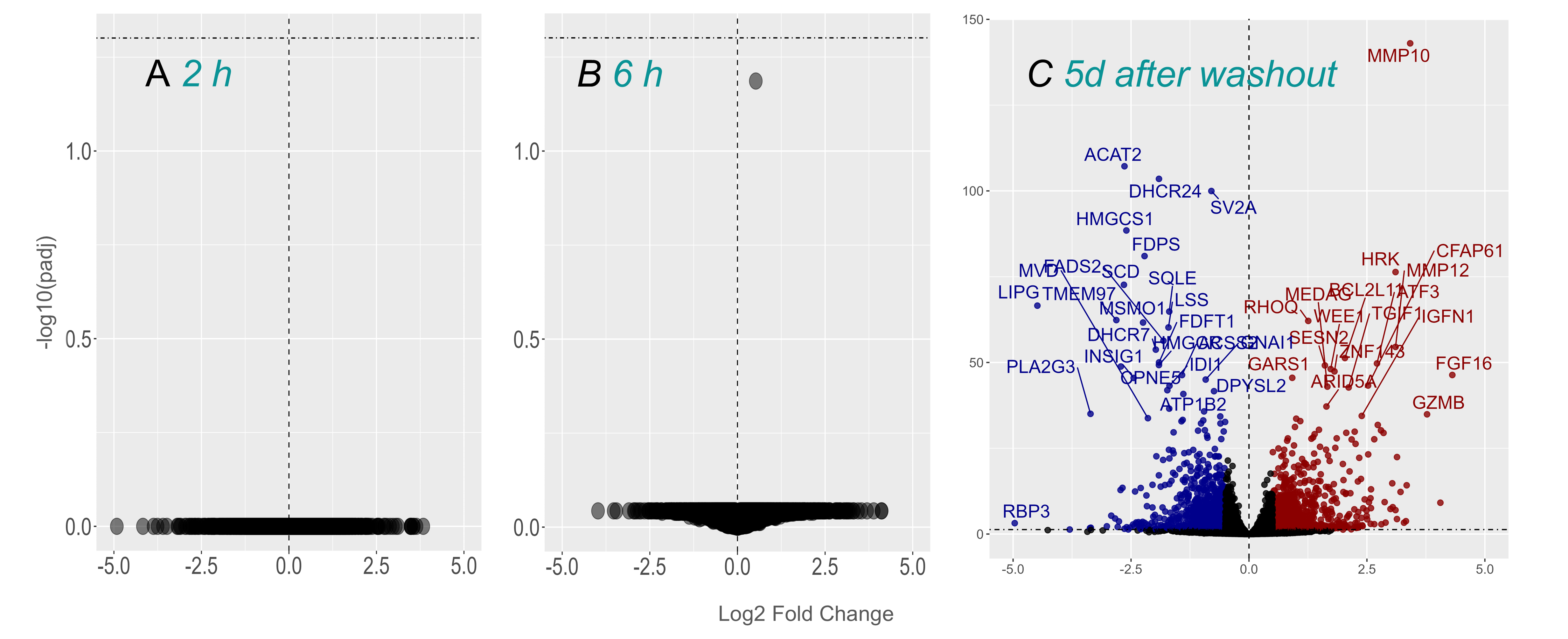

Supplement: Supplementary file 6 — Supplementary Figure 5. [file 41419_2026_8445_MOESM6_ESM.tif]

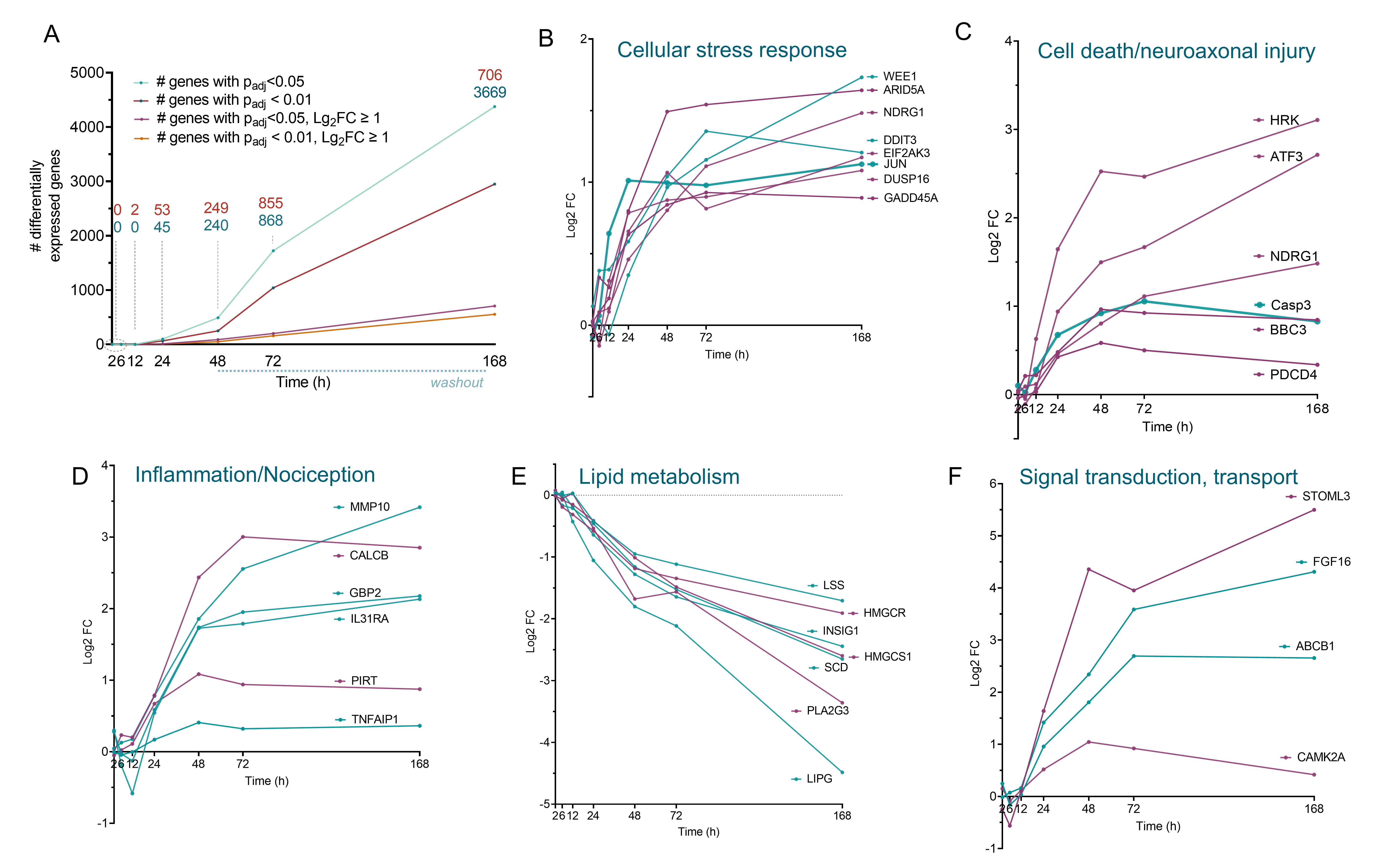

Supplement: Supplementary file 7 — Supplementary Figure 6. [file 41419_2026_8445_MOESM7_ESM.tif]

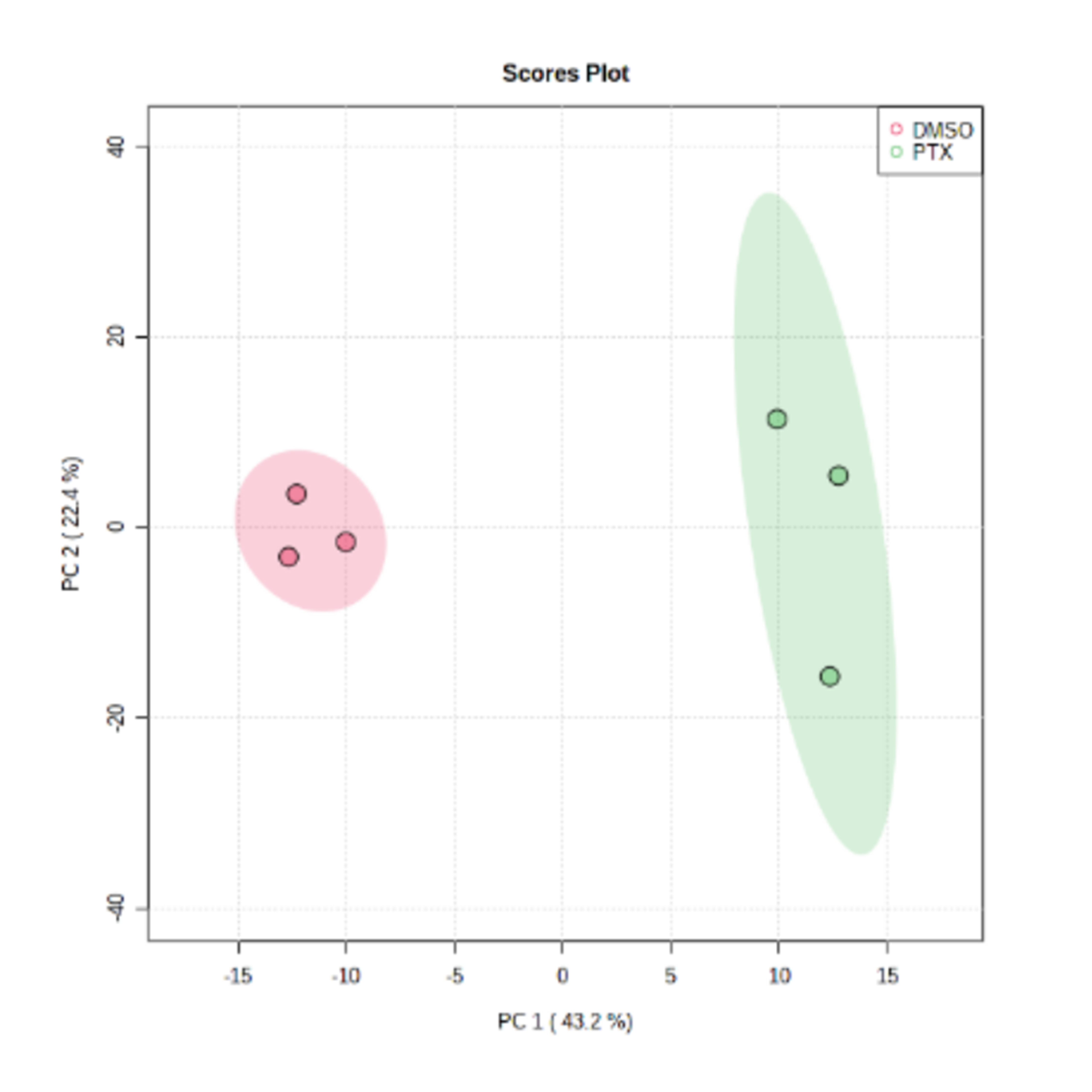

Supplement: Supplementary file 8 — Supplementary Figure 7. [file 41419_2026_8445_MOESM8_ESM.tif]

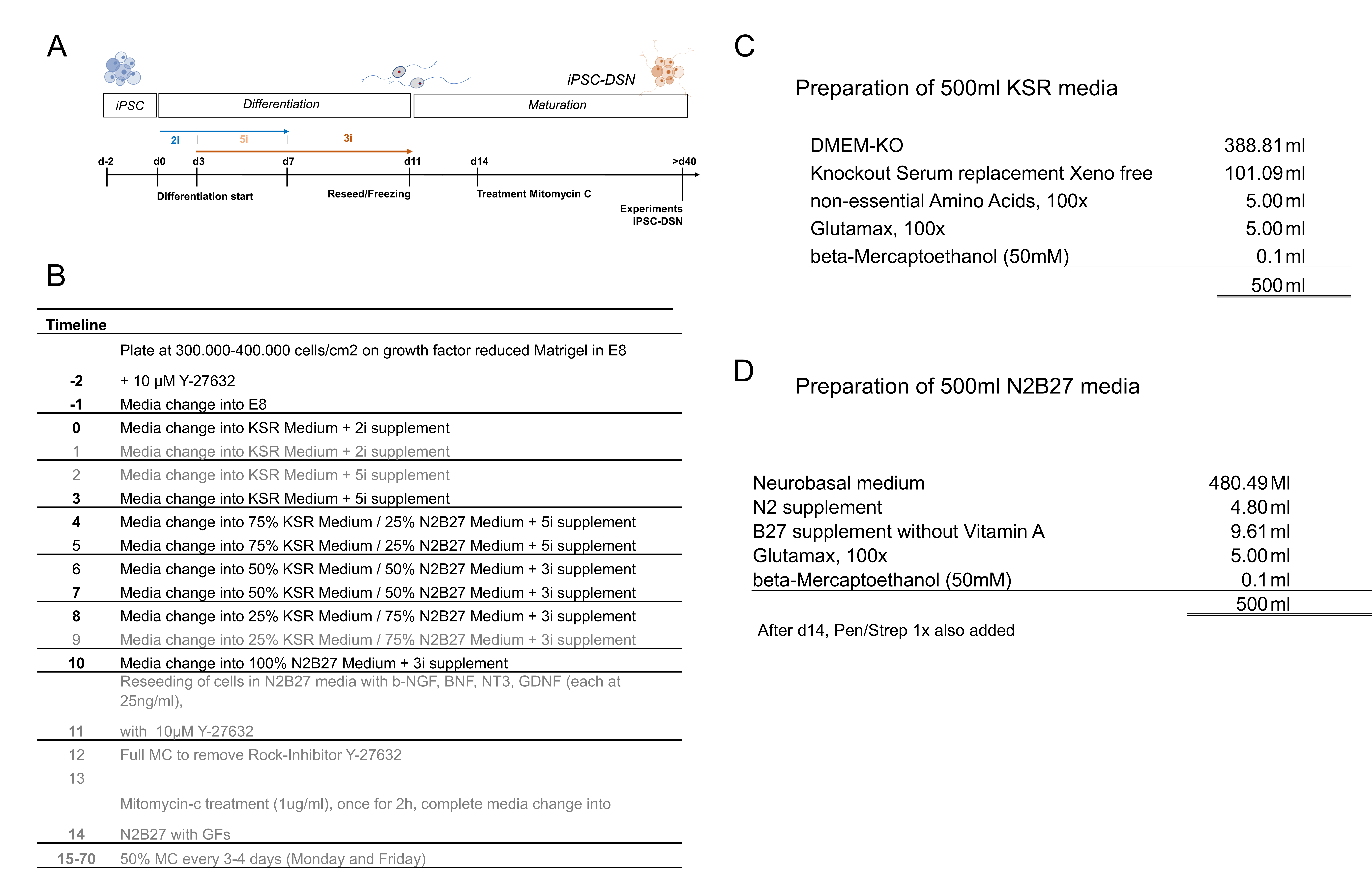

Supplement: Supplementary file 9 — Supplementary Figure 8. [file 41419_2026_8445_MOESM9_ESM.tif]
